# Evolutionary Relationships among *Chlamydophila abortus* Variant Strains Inferred by rRNA Secondary Structure-Based Phylogeny

**DOI:** 10.1371/journal.pone.0019813

**Published:** 2011-05-24

**Authors:** Victoria I. Siarkou, Alexandros Stamatakis, Ilias Kappas, Paul Hadweh, Karine Laroucau

**Affiliations:** 1 Laboratory of Microbiology and Infectious Diseases, Faculty of Veterinary Medicine, Aristotle University of Thessaloniki, Thessaloniki, Greece; 2 The Exelixis Lab, Scientific Computing Group, Heidelberg Institute for Theoretical Studies, Heidelberg, Germany; 3 Department of Genetics, Development and Molecular Biology, School of Biology, Aristotle University of Thessaloniki, Thessaloniki, Greece; 4 Bacterial Zoonoses Unit, French Agency for Food, Environmental and Occupational Health and Safety, Maisons-Alfort, France; University of California San Francisco, University of California, Berkeley, and the Children's Hospital Oakland Research Institute, United States of America

## Abstract

The evolutionary relationships among known *Chlamydophila abortus* variant strains including the LLG and POS, previously identified as being highly distinct, were investigated based on rRNA secondary structure information. PCR-amplified overlapping fragments of the 16S, 16S-23S intergenic spacer (IS), and 23S domain I rRNAs were subjected to cloning and sequencing. Secondary structure analysis revealed the presence of transitional single nucleotide variations (SNVs), two of which occurred in loops, while seven in stem regions that did not result in compensatory substitutions. Notably, only two SNVs, in 16S and 23S, occurred within evolutionary variable regions. Maximum likelihood and Bayesian phylogeny reconstructions revealed that *C. abortus* strains could be regarded as representing two distinct lineages, one including the “classical” *C. abortus* strains and the other the “LLG/POS variant”, with the type strain B577^T^ possibly representing an intermediate of the two lineages. The two *C. abortus* lineages shared three unique (apomorphic) characters in the 23S domain I and 16S-23S IS, but interestingly lacked synapomorphies in the 16S rRNA. The two lineages could be distinguished on the basis of eight positions; four of these comprised residues that appeared to be signature or unique for the “classical” lineage, while three were unique for the “LLG/POS variant”. The U277 (*E. coli* numbering) signature character, corresponding to a highly conserved residue of the 16S molecule, and the unique G681 residue, conserved in a functionally strategic region also of 16S, are the most pronounced attributes (autapomorphies) of the “classical” and the “LLG/POS variant” lineages, respectively. Both lineages were found to be descendants of a common ancestor with the Prk/Daruma *C. psittaci* variant. Compared with the “classical”, the “LLG/POS variant” lineage has retained more ancestral features. The current rRNA secondary structure-based analysis and phylogenetic inference reveal new insights into how these two *C. abortus* lineages have differentiated during their evolution.

## Introduction

*Chlamydophila abortus* is an intracellular bacterium that is able to efficiently colonize the placenta of several mammals causing abortion and premature birth of stillborn or weak neonates [1]–[4]. This pathogen is endemic among small ruminants and represents a zoonotic pathogen. Pregnant women exposed to infected animals have the risk of spontaneous abortion or even a life-threatening disease [4]. *Chlamydophila abortus* is classified as a member of the family *Chlamydiaceae* which currently encompasses the two genera *Chlamydia* and *Chlamydophila*, subdivided into three (*C. muridarum, C. suis, C. trachomatis*) and six (*C. abortus, C. caviae, C. felis, C. pecorum, C. pneumoniae, C. psittaci)* species, respectively [2], [5]. Genetic analyses indicate that *C. abortus* has evolved from *Chlamydophila psittaci*, which also constitutes a zoonotic pathogen associated primarily with avian chlamydiosis [2], [6], [7].

Studies using different phenotypic and molecular approaches suggest that *C. abortus* is a homogeneous species and includes strains sharing distinctive inclusion morphology and antigenic profile, and nearly 100% sequence conservation in the ribosomal and *ompA* genes [2], [8]–[11]. However, two homologous strains, namely LLG and POS, isolated in Greece from an aborted goat and ewe, respectively [12], were considerably different among other *C. abortus* strains prevailing in the same area and were characterized as variants on the basis of unique inclusion morphology, differences in polypeptide profiles, non-reactivity with monoclonal antibodies against immunodominant *C. abortus* antigens, diversity of 23S domain I rRNA and *ompA* sequences, and different behavior in cell cultures and mouse model protection experiments [12]–[16]. In a recent study using multiple-locus variable number tandem repeat (VNTR) sequences, the LLG and POS strains were identified as the most divergent ones among other *C. abortus* strains, constituting a distinct genotype, in particular for the *pmp5E* and *hctB* loci involved in establishing the immunodominant and structural proteins, respectively [17]. Moreover, sequencing of the LLG RFLP-fragments of the plasticity zone, a region of extensive gene differences between *Chlamydiaceae* species, revealed considerable differences in the pseudogene content [18]. Similar variation in biological and/or genotypic characteristics, albeit to a lesser extent, has also been observed among other *C. abortus* strains [12]–[15], [17], [19].

The previous studies have raised novel questions regarding the actual evolutionary relationships of the variant *C. abortus* strains that share a common geographical origin. To this end, the information content of rRNA genes is especially useful for providing a solid framework for the assessment of evolutionary changes in lineages [20]–[24]. Moreover, rRNAs are functionally constrained structure mosaics ranging from highly conserved to more variable ones, with varying evolutionary rates among secondary structure elements [20], [25]–[29]. In the present study, PCR-amplified overlapping fragments of the “ribosomal operon” derived from *C. abortus* variant strains, including the LLG and POS, were subjected to cloning and sequencing. We firstly focused on the 16S rRNA and 16S-23S intergenic spacer (IS) genes since the 23S rRNA domain I gene sequences for the respective strains had been previously determined [12]. We aimed at investigating the pattern and distribution of signature or unique nucleotide residues in rRNA molecules among *C. abortus* variant strains as well as on inferring their phylogenetic relationships based on rRNA secondary structure. The information gained may contribute to a more thorough understanding of the mode of molecular evolution in *C. abortus*.

## Materials and Methods

### Chlamydial strains and DNA preparations

The *C. abortus* strains FAS, FAG, VPG, LLG and POS, all isolated in Greece from aborted sheep or goat fetuses [12], were used in the present study. All strains have been previously described on the basis of inclusion morphology, antigenic and molecular diversity [12], [15], and recently classified into three distinct VNTR genotypes [17]. Whole genomic DNAs were extracted (NucleoSpin tissue kit; Macherey-Nagel) from the second passage of the original isolates, propagated in yolk sac of embryonated chicken eggs, so as to represent fresh clinical isolates and not laboratory-adapted strains.

### PCR amplification, cloning and sequencing of rDNAs

PCR amplifications resulting in four overlapping PCR-amplified rDNA fragments were conducted as previously described [2], [5], [30] with some modifications. Briefly, two PCR amplifications intended for amplifying the entire 16S rDNA were performed by using the primer pairs 16SFor/16SIGR and 16SF/16SR, yielding fragments encoding the 16S signature sequence and nearly the full-length 16S rRNA, respectively. Two additional PCR amplifications were performed by using the primer pairs 16SF2/23R and 16SF2/23SIGR for amplifying the 16S-23S IS flanked by 16S and 23S segments, including the 23S signature sequence (domain I). A schematic representation of the four overlapping PCR-amplified rDNA fragments as well as the primer pairs used for the PCR amplifications with the respective annealing temperatures are available as supporting material (Figure S1, Table S1).

The rDNA sequences were determined by both direct PCR sequencing and sequencing of cloned products. Initially, purified (NucleoSpin Extract II kit; Macherey-Nagel) PCR products from two separate PCR reactions for each fragment for each strain were sequenced (ABI 3730XL, Macrogen) on both strands using the respective PCR primers and an internally designated primer (Table S1). In addition, clone libraries of purified PCR products obtained from a separate series of PCR reactions were constructed by ligation into the pCR2.1 vector (TA cloning kit; Invitrogen) and transformation, by heat shock, into *E. coli* XL-1 Blue (Stratagene). Blue-white screening of transformants [31] was performed on LB agar containing ampicillin (100 µg/ml) and top spread with IPTG (0.5 mM) and X-Gal (80 µg/ml). From each clone library, ten white colonies were picked randomly and screened by PCR for the presence of rDNA inserts. Subsequently, four independent clones were selected and sequenced following extraction of the recombinant plasmid DNA (NucleoSpin Plasmid QuickPure kit; Macherey-Nagel). The T7 promoter and the M13R-pUC primer flanking the multiple cloning site of pCR2.1 DNA were used to sequence both DNA strands. On the basis of the four PCR-amplified rDNA fragments, which overlapped one another (Figure S1), as well as the PCR amplification and sequencing strategies, the corresponding sequences that were obtained each had a 6x up to 12x read coverage.

### Secondary structure-based rDNA sequence analysis

The obtained sequences were initially compared with the public sequences using the BLAST program at NCBI (http://www.ncbi.nlm.nih.gov/). The overlapping rDNA sequences were initially aligned together using CLUSTAL X 1.83 [32], and then for each rDNA locus multiple sequence alignments with reference sequences downloaded from the NCBI database were computed. In order to construct sequence alignments on the basis of 16S and 23S rRNA secondary structure modelling, sequences were automatically aligned by SINA, as implemented in the SILVA SSU and LSU rRNA database project (http://www.arb-silva.de/; [33]). The 16S rDNA sequences were also aligned via the NAST aligner (http://greengenes.lbl.gov/; [34]). As a control for the effects of using secondary structure-based alignment algorithms, specific data for rRNA sequences were used from the Comparative RNA Web (CRW) relational database management system (RDBMS) (http://www.rna.ccbb.utexas.edu/; [35]). Helix numbering for the 16S and 23S rRNA secondary structures followed the respective reference sequence numbering system (*E. coli* GenBank acc. no. J01695) according to CRW [35]. Nucleotide frequency and conservation data were also derived from the CRW site.

### Phylogeny reconstruction

Phylogeny reconstruction was performed using maximum likelihood (ML) and Bayesian Inference (BI) approaches. It has repeatedly been demonstrated [20], [23], [36], [37] that likelihood-based approaches (ML and BI) are able to recover the true tree more frequently than parsimony or distance-based (e.g., Neighbor-Joining) approaches. For ML-based analyses the most recent version 7.2.6 of RAxML (http://wwwkramer.in.tum.de/exelixis/software.html; [38]) was used as it has been shown to perform best among all other methods tested by Price et al. [37]. For BI-based analyses, MrBayes version 3.1.2 was used (http://mrbayes.csit.fsu.edu/; [39]).

RAxML under the GTR+Gamma substitution model [40] (see RAxML manual at http://icwww.epfl.ch/~stamatak/index-Dateien/Page443.htm) was used to infer 1,000 bootstrap replicates and to conduct 50 ML searches on the original alignment using 50 distinct randomized stepwise addition parsimony trees. The respective RAxML options were used to draw bootstrap support (BS) values onto the best-scoring ML tree obtained on the original alignment as well as to compute majority-rule consensus trees from the collections of bootstrap replicates and ML trees. Bayesian inference was also conducted under the GTR+Gamma model using two independent runs with four Metropolis-Coupled Markov Chains each. Ten million generations were performed for each region using default priors with trees sampled every 100^th^ generation (burnin set to 10,000 generations) to obtain posterior probabilities.

Dendroscope (http://www-ab.informatik.uni-tuebingen.de/software/dendroscope/; [41]) and TreeGraph 2 (http://treegraph.bioinfweb.info/; [42]) were used for tree visualization and manipulation.

## Results and Discussion

### Nucleotide sequences of the rRNA molecules

Directly obtained sequences of the rDNA fragments produced by the two separate PCR reactions and the corresponding sequences of the separately produced independent clones (including all overlapping segments in each case) were 100% identical. This resulted in an unambiguous determination of the entire lengths of the 16S, 16S-23S IS, and 23S domain I rRNA genes for each *C. abortus* strain examined. Sequence identity among PCR-amplified rDNAs and cloned products for each strain indicated that only one gene from each rRNA locus is present in *C. abortus*, which is in agreement with previous findings [18].

However, the rDNA sequences obtained from the five *C. abortus* strains under investigation (GenBank accession numbers EF486853-EF486857) exhibited differences. Regarding the 16S rRNAs, the comparison revealed the presence of two sequence variants differing by four single nucleotide variations (SNVs). More precisely, the nucleotide sequences of LLG and POS strains were identical to each other but differed from those of the FAS, FAG, and VPG strains by the presence of nucleotides A, C, G, and G instead of G, U, A, and A at positions 80, 277, 396, and 681 (according to the *E. coli* numbering system) [35], respectively. Interestingly, at positions 277 and 396 the LLG and POS variants shared identical nucleotides with the *C. abortus* type strain B577^T^, while the sequences of FAS, FAG, and VPG strains were found to be identical to those of other classical (well-established) strains of this species (i.e., S26/3, EBA, EAE, and OEA).

The 16S-23S IS rRNA sequences obtained from the *C. abortus* strains were identical in all but one SNV. This variation, nucleotide U instead of C, located at position 79 (according to the *C. abortus* type strain B577^T^ sequence; GenBank acc. no. U68445) was detected in the LLG and POS sequences.

The sequencing results of the 23S domain I region of the current study confirmed previous ones [12] regarding the presence of four SNVs. Three of them, namely A, C, and G instead of G, U, and A located at positions 152, [181-182], and 273 (*E. coli* numbering; square brackets indicate that *E. coli* lacks the corresponding position), respectively, were identified in the LLG and POS sequences, and one, U instead of C at position 547, was identified in the FAG and VPG sequences.

To evaluate the importance of these sequence differences (discussed below), alignments of 16S and 23S rRNA sequences of 71 and 67 strains, respectively, belonging to *C. abortus* and other related species were constructed on the basis of the corresponding secondary structure information. Alignment segments corresponding to the structural elements bearing the SNVs are available as supporting material (Figure S2A and S3A). An alignment of 16S-23S IS sequences of 57 chlamydial strains was constructed on the basis of the primary structure (Figure S4A), since the intergenic spacer is the most variable region throughout the rRNA operon and an analogous secondary structure-based reference numbering system does not exist. In Table 1 we provide the variant residues found in *C. abortus* compared with related taxa.

**Table 1:** Nucleotide variations in rRNA molecules of *C. abortus* strains compared with related taxa and respective nucleotide frequency data within the domain *Bacteria*.

Organisms	Base or base pair in								
	16S rRNA				16S-23S IS	23S rRNA (domain I)			
	80:89^a^	247:277	45:396	681:709	79	152:174	[181–182]	273:364	547
*Chlamydophila abortus* B577^T^ VR-656 (D85709, U68445)	G:U	G:**C**	U:**G**	A:U	C	G:U	U	A:U	C
*C. abortus* LLG (EF486856)	**A**:U	G:**C**	U:**G**	**G**:U	**U**	**A**:U	**C**	**G**:U	C
*C. abortus* POS (EF486857)	**A**:U	G:**C**	U:**G**	**G**:U	**U**	**A**:U	**C**	**G**:U	C
*C. abortus* FAS (EF486853)	G:U	G:U	U:A	A:U	C	G:U	U	A:U	C
*C. abortus* FAG (EF486854)	G:U	G:U	U:A	A:U	C	G:U	U	A:U	**U**
*C. abortus* VPG (EF486855)	G:U	G:U	U:A	A:U	C	G:U	U	A:U	**U**
*C. abortus* S26/3 (CR848038)	G:U	G:U	U:A	A:U	C	G:U	U	A:U	C
*C. abortus* EBA (U76710)	G:U	G:U	U:A	A:U	C	G:U	U	A:U	C
*C. abortus* EAE (Z49871), A22 (U68444)	G:U	G:U	U:A	A:U	C	G:U	U	A:U	C
*C. abortus* OEA (Z49872), OSP (U68446)	G:U	G:U	U:A	A:U	C	G:U	U	A:U	C
*Chlamydophila psittaci* 6BC^T^ VR-125 (U68447)^b^	G:U	G:C	U:G	A/g:U	C	A:U	C	A:U	A/c
*Chlamydophila caviae* GPIC^T^ VR-813 (AE015925)^b^	A:U	G:C	U:G	A:U	C	A:U	C	A:U	C
*Chlamydophila felis* FP Baker^T^ VR-120 (D85701, U68457)^b^	G:U	G:C	U:G	A:U	C	A:U	C	A:U	C
Chlamydophila pecorum *E58^T^ VR-628 (D88317, U68433)* b	G:U	G:C	U:G	A:U	C	A:U	A	A:U	C
*Chlamydophila pneumoniae* TW-183^T^ VR-2282 (L06108, U76711)^b^	G/a:U	G:C	U:G	A:U	C	A:U	C	A:U	C
*C. pneumoniae* N16 (U68426)	G:U	G:C	U:G	A:U	C	A:U	C	A:U	C
*C. pneumoniae* LPCoLN (FJ236984)	G:U	G:C	U:G	A:U	na	na	na	na	na
*Chlamydia trachomatis* A/Har-13^T^ VR-571B (D89067, U68438)^b^	U:G/A	G:C	U:G	A:U	U	G:C	C	A/g:U	A
C. trachomatis *L2/434/BU VR-902B (U68443, U68443)* b	U:A	G:C	U:G	A:U	U	G:C	C	A:U	A
Chlamydia muridarum *MoPn^T^ VR-123 (D85718, U68436)* b	U:G	G:C	U:G	A:U	C	G:C	C	G:U	A
*Chlamydia suis* S45^T^ VR-1474 (U73110)^b^	U:A	G:C	U:G	A:U	C	G:C	C	A:U	A
*Parachlamydiaceae* sp. Bn9^T^ VR-1476 (Y07556, AF193069, Y07555)^b^	−	G:C	U:G	A:U	C	U:A/C:G	U/C	G:C/A:U	U/A
*Waddliaceae* sp. WSU 86/1044^T^ VR-1470 (AF042496)^b^	G:U	G:C	U:G	C/A:U	C	U:A	G	G:U/G:C	A
*Simkaniaceae* sp. Z^T^ VR-1471 (U68460)^b^	A:U/−	G:C	U:G	A:U	A	C:G/U:A	U/−	G/A:U	U
*E. coli* (J016950)	A:U	G:C	G:C	A:U		A:U	−	G:C	A
**Domain** ***Bacteria***									
Base pair frequencies based on 16S & 23S rRNA models^c^	G:C 32.5 C:G 18.9 U:A 18.1 A:U 12.1 G:U 5.2 U:G 1.2 Gap 9.7	G:C 99.2 C:G ---- U:A ---- A:U 0.1 G:U 0.4 U:G ---- Gap ----	G:C 27.0 C:G 5.3 U:A 37.2 A:U 0.8 G:U 0.2 U:G 25.2 Gap ----	G:C 10.5 C:G 16.7 U:A 40.6 A:U 31.2 G:U 0.1 U:G ---- Gap ----		G:C 12.6 C:G 37.0 U:A 10.4 A:U 27.0 G:U 6.3 U:G 0.4 Gap 1.5		G:C 28.2 C:G 19.5 U:A 21.7 A:U 7.2 G:U 4.7 U:G ---- Gap 15.6	
Single base frequencies based on 16S & 23S rRNA models^c^	A 14.05 G 37.93 C 19.75 U 19.75	A 0.00 G 0.10 C 99.27 U 0.59	A 37.42 G 30.29 C 31.05 U 1.20	A 31.51 G 10.63 C 17.00 U 40.77		A 27.78 G 19.63 C 37.78 U 13.70		A 8.63 G 33.81 C 21.58 U 21.94	A 53.76 G 9.32 C 16.13 U 20.79

a Nucleotide positions of the 16S rRNA and 23S domain I rRNA are given according to *E. coli* J01695 secondary structure numbering system [35]; position of 16S-23S Intergenic Spacer based on primary structure is given according to the *C. abortus* type strain B577^T^ sequence (U68445). Positions of the single nucleotide variations (SNVs) are indicated in boldface. The [181–182] position represents “insertion” position.

b More than two or three accession numbers for each species or family including the type strain (^T^), were analyzed (the majority of analyzed strains and their accession numbers are given in the Figures S2, S3 and S4); lower-case letters denote residues found only in one of the examined strains; “na”, not available data; “−”, nucleotide gap in rRNA sequence comparison.

c Dataset from http://www.rna.ccbb.utexas.edu/SAE/2A/nt_Frequency/

### Secondary structure-based nucleotide analysis of the rRNA molecules

As an additional means of assessing genetic relatedness, and in order to check whether the observed SNVs are located within particular evolutionary variable or conserved regions of rRNAs potentially supporting phylogenetic groupings, we conducted comparisons with other available chlamydial sequences on the basis of their secondary structure.

#### 16S rRNA analysis

All four SNVs corresponding to positions 80, 277, 396, and 681 occurred in stem regions (base-pairing regions) of the secondary structure helices H61(61-82/87-106), H240(240-259/267-286), H39(39-46/395-403), and H673(673-690/697-717) (helix numbering according to Comparative RNA Web (CRW) site; [35]), respectively. These SNVs did not result in a nucleotide substitution in the complementary position of the stem (Table 1, Figure S2A). Based on comparative sequence analyses (data available at CRW site; see Table 1), SNV at position 277 corresponded to a highly conserved residue throughout domain *Bacteria* (more than 98%), SNVs at positions 396 and 681 corresponded to less highly conserved residues (less than 80%), whereas SNV at position 80 was found to exist within an evolutionary variable region. Interestingly, at location 247∶277 the LLG/POS variant, as well as the *C. abortus* type strain B577^T^, exhibited the base pair G:C occurring in most members of the domain *Bacteria* (99.2%) and also shared by all *Chlamydiales* species, but not by the remaining *C. abortus* strains. The latter, possessed a G:U base pair rarely occurring at this location throughout domain *Bacteria* (0.4%) (Table 1). Similarly, at location 45∶396, the LLG/POS variant and the B577^T^ strain presented the base pair U:G, shared with all *Chlamydiales* species but not the remaining *C. abortus* strains which presented the equally common U:A base pair. Notably, the feature G:U at location 681∶709 only found in the LLG/POS variant, was not present in the 16S rRNA molecule of any other species of the order *Chlamydiales*, with only one *C. psittaci* strain exception, and was also rarely found among bacteria (0.1%) (Table 1). Another attribute of the LLG/POS variant was the base pair A:U at location 80∶89, not present in the vast majority of the *Chlamydiales* (Table 1).

In each of the four 16S rRNA variations a transitional substitution was observed so that a G:U type base pairing, at no “dominant” G:U type sites [25], [43], was interchanged with canonical base pairing (A:U and G:C types) or vice versa (Table 1, Figure S2A), suggesting a strong selection for pyrimidine:purine base pairing. This sequence variation may not be necessarily involved in any obvious structural features that serve a specific binding mechanism; however, this depends on residue conservation [44] as well as on the functional significance of the structure element in which the variation occurs. Strikingly, a new phenotype mutant had been generated by a single C→U transition at the “universally conserved residue” G11:C23 (99.1%, three Phylogenetic domains [3P]) of the 5′ terminal pseudoknot H9 helix of 16S rRNA [45]. In the present case, the “conserved residue” G247:C277 (99.2%, domain *Bacteria*; 87.3%, 3P), flanked by an asymmetric internal loop inside the highly conserved H240 helix of the 5′ domain [46] (CRW site dataset; Figure S2A), could be selected as more stable than G:U (0.4%, domain *Bacteria*; 0.3%, 3P) in the context of a loop-closing base pair [25]. Interestingly, location 247∶277 and the region around it is important for recognition by the ribosomal protein S17, which strongly protects the region, binds, and stabilizes the H240 helix near the central junction [47]–[50]. The feature G247:U277 observed in most of *C. abortus* strains could function as a recognition signal for protein binding, which through stabilizing of the created “wobble” pair and alleviating what would otherwise be a deleterious condition could facilitate the evolutionary replacement of the base pair [28]. Additionally, it has been proven that G:U pairs could enhance stability as closing base pairs in specific contexts [43]. Some of the H240 positions also constitute parts of the S20 binding site [49], [50] and, genetic studies have shown that substitutions and deletions at these sites could abolish binding of S20 to 16S rRNA [51]. Generally, “conserved positions” throughout 3P represent the preservation of specific structural elements, which presumably act as scaffolds to provide the critical orientations of highly conserved residues in three-dimensional space [44]. Indeed, the “conserved position” 396 occurring in the interior of the H39 helix is flanked by a single bulge “universally conserved” adenosine residue (Figure S2A) [25], [46], [52], known as a binding site of the S4 protein, which is essential for the stability of the rRNA tertiary interactions [47], [49], [53], [54]. At position 396 (nucleotide 5′ of bulge), purines (A or G) are almost equally common (Table 1) suggesting that there is no particular pressure to favor one or the other base adjacent to the bulge. Structural reasons for the selection of U45:A396 instead of U45:G396 at this site are thus not apparent. The “conserved position” 681 is an intrahelical site of the helix H673 which is a functionally strategic region of the 16S rRNA [46], [55]. The location 681∶709 is found in the vicinity of “universally” or “highly conserved” residues known to be involved in heterodimer S6 and S18 protein bindings [47], [48], [56] or residues involved in S11 protein binding, which is essential for stabilizing the 16S rRNA central domain folding [47], [48], [57], [58] and specific E-site tRNA interactions [44], [59]. At this site, the high incidence of the canonical type base pairs (Table 1) could reflect its functional implication, whereas the rarity of the “wobble” G681:U709 pair could reflect a likely alteration in the respective interdependent interactions; the irregularity, probably caused by the G:U residue, may be a signal for specific protein bindings [28], [55], [60]. Remarkably, interdependencies of protein binding in the assembly of the central domain are similar but not identical among different microorganisms [57]. Finally, location 80∶89 is situated in a highly variable area corresponding to the “non-conserved region” (residues 79–100) of the H61 helix (Figure S2A), one of the most informative or discriminating regions for closely related organisms [20], [25], [44]. This area exhibits genetic-group specificity for the order *Chlamydiales* (variable region I of the 16S rRNA signature sequence) [2], [61] with intraspecific sequence variation also occurring within chlamydial species such as the equine-type (strain N16) of *C. pneumoniae* and the E, F, and L2 types of *C. trachomatis* (Figure S2A). The feature A80:U89 exhibited by the LLG and POS *C. abortus* variant strains also occurred in *C. caviae* as well as in *C. pneumoniae* and *S. negevensis* single strains, but without the corresponding variable region being entirely similar (Table 1, Figure S2A). Character homology in variable regions is not necessarily indicated by sequence identity or similarity [20] (discussed below).

#### 23S domain I rRNA analysis

Two of three SNVs observed in the LLG/POS variant, corresponding to positions 152 and 273 (*E. coli* numbering), occurred in stem regions of the helices H150(150–158/168–176) and H271(271–297/341–366), respectively. Similarly to the 16S rRNA variations, none of these SNVs has resulted in a compensatory substitution, but G:U pairing was interchanged with canonical A:U pairing or vice versa (Table 1, Figure S3A). Both H150 and H271 helices comprise particularly variable regions that are phylogenetically informative for the identification and taxonomy of bacterial pathogens [26], [62], [63]. The SNV at position 152, corresponding to a conserved residue (less than 80%, domain *Bacteria* & 3P), was adjacent to a “non-conserved region” (residues 153–173) of the H150 helix, whereas the SNV at position 273 occurred within the “non-conserved region” (270–297/353–369) of the H271. At location 152∶174 the LLG/POS *C. abortus* variant exhibited the base pair A:U, also shared by all *Chlamydophila* species but not the remaining *C. abortus* strains. The latter, possessed a G:U base pair characterized by low frequency at this location throughout domain *Bacteria* (6.3%) (Table 1). In contrast, the base pair G:U at location 273∶364 found in the LLG/POS variant, was not present in any *Chlamydophila* species but only in isolated cases within the order *Chlamydiales* and with low frequencies among bacteria (4.7%) (Table 1). The third SNV of the LLG/POS variant, corresponding to position [181–182], occurred in an unpaired position immediately adjacent to the “universally conserved” adenosine residue (position 182) at the 3′ end of the multi-stem loop (Figure S3A) [52]. At this position, the LLG/POS variant presented the residue C shared by most *Chlamydiales* species while the remaining *C. abortus* strains presented the residue U. It is not apparent how the residue U prevailed and how it may affect the conformation of the AG opposition at the end of the H183 helix [64]. Finally, the SNV observed in 23S rRNA domain I of the FAG/VPG variant at position 547 occurred in the hairpin-loop of the H533 helix and corresponded to a conserved residue across domain *Bacteria* (less than 80%). It is worth noting that the sequence corresponding to residues 543–552 is a “non-conserved region” throughout 3P (data available at CRW site) and often presents remarkable intraspecific diversity within domain *Bacteria* [65]. The residue U found in the FAG/VPG variant, albeit frequently present among bacteria (20.79%), rarely occurs among *Chlamydiales* species (Table 1).

#### 16S-23S IS analysis

The SNV detected in the LLG/POS variant, corresponding to position 79 (according to *C. abortus* type strain B577^T^ sequence; acc. no. U68445, Figure S4A) was found to occur in a stem region which is predicted to be formed between the chlamydial spacer and a complementary segment of 16S promoter sequence [66]. In this context, the variant residue could be paired with a residue immediately adjacent to the 3′ end of the promoter P2-10 sequence (Figure S4B), a region of high functional stringency and conservation [66]–[68]. However, it is worth noting that residue U found in LLG/POS variant was also shared by *C. trachomatis* strains within the order *Chlamydiales* (Table 1).

### Phylogenetic analysis

**Figure 1:**
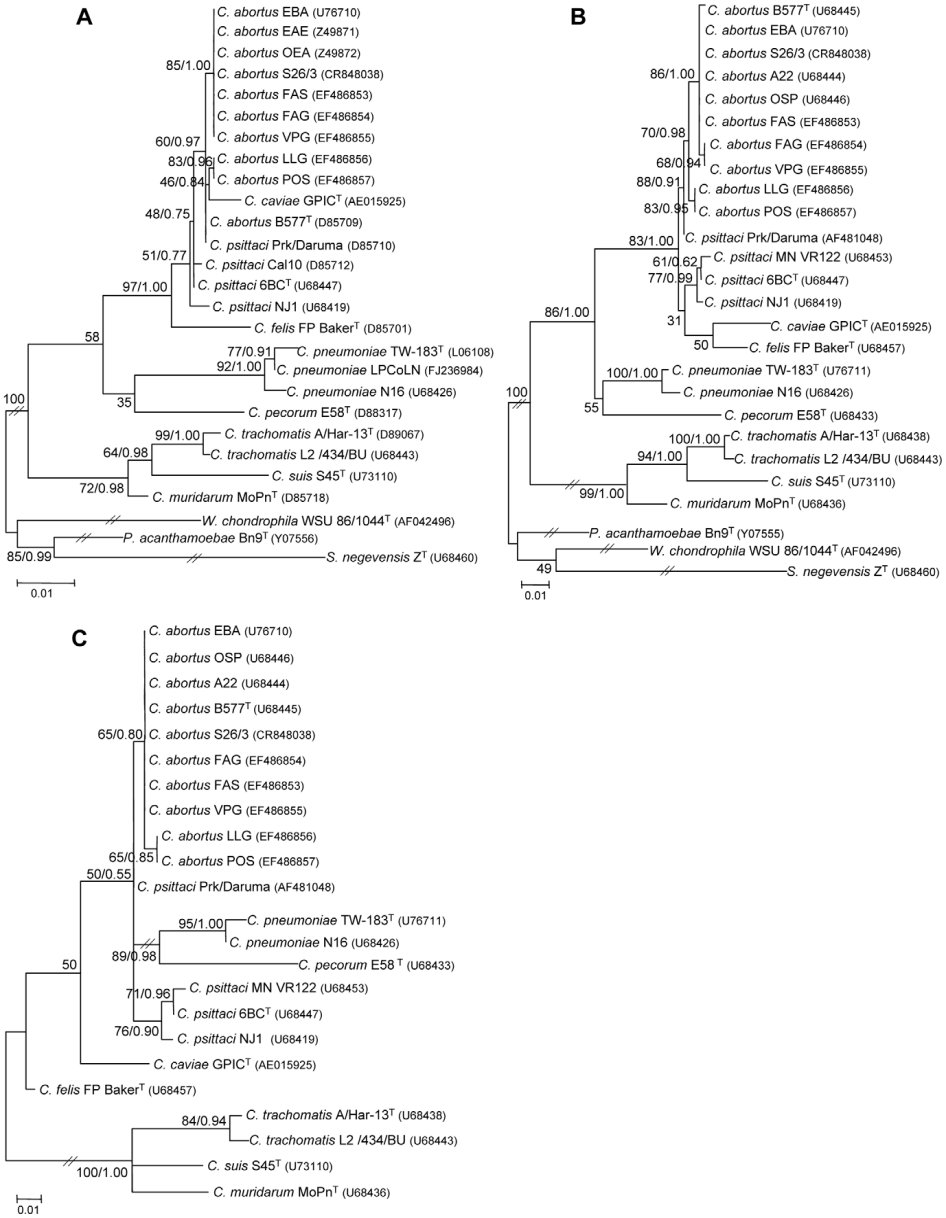
Best-scoring maximum likelihood trees based on 16S (A), 23S domain I (B), and 16S-23S IS (C) rRNA chlamydial sequences. Full-length sequences of *C. abortus* variant strains and representatives from other *Chlamydiaceae* species were used. The type strains of other families within the order *Chlamydiales* were included as outgroups in the 16S and 23S rRNA trees. The trees were reconstructed using RAxML 7.2.6 [38]. The 16S and 23S rRNA trees were generated on the basis of secondary structure alignments created by SINA (SILVA SSU and LSU rRNA database project; [33]) while the 16S-23S IS tree was based on primary structure alignment computed using CLUSTAL X 1.83 [32]. Numbers on branches are support values to clusters on the right of them. Maximum likelihood bootstrap percentages and Bayesian posterior probabilities are included for clades that were consistently recovered using both phylogenetic methods (otherwise only bootstrap values are shown). Bayesian consensus trees are available as supporting material (Figure S5). Accession numbers for sequences retrieved from GenBank as well as for the sequences generated in this study are shown in parentheses. The mark//indicates that branches were shortened for visualization purposes.

Phylogenetic trees were constructed using a subset of 27 or 26 full-length sequence alignments of the 16S, 23S domain I, and 16S-23S IS rRNAs. In particular, all available *C. abortus* sequences as well as representatives (type or reference strains) from all other hitherto defined species within *Chlamydiaceae*, including intraspecific variants, were employed to elucidate the evolutionary relationships of the *C. abortus* variants. The type strains of the families *Parachlamydiaceae*, *Waddliaceae*, and *Simkaniaceae*, phylogenetically positioned in the order *Chlamydiales*, were used as outgroups in the 16S and 23S rRNA trees. These outgroups were not used in 16S-23S IS tree due to their limited sequence identity with *Chlamydiaceae* and therefore the difficulty to align (Figure S4A). The best-scoring likelihood trees for 16S, 23S, and 16S-23S IS inferred by RAxML under the GTR+Gamma substitution model, are shown in Figure 1 (A, B and C). Branch support values (in congruent arrangements) for each of the two different approaches used (bootstrap support values from ML analyses, BS; Bayesian posterior probabilities, PP) are also indicated.

#### 16S rRNA analysis

The overall topology of the best-scoring ML tree inferred with RAxML was consistent with previously determined phylogenies using other algorithms [2], [6], [61] (Figure 1A). The tree constructed by the Bayesian approach differed in that it showed *C. pneumoniae* to form a distinct line of descent, separated from those of *C. pecorum* and other *Chlamydiaceae* taxa, resulting in an overall topology resembling the previously published phylogeny by Pettersson et al. [69] (Figure S5A). However, the Bayesian and ML trees were congruent with respect to the following characteristics:

- A well-supported clade that contained *C. felis*, *C. psittaci*, *C. caviae*, and *C. abortus* was present in both analyses (BS, 97%; PP, 1.00).
- Interestingly, both phylogenetic approaches recovered the *C. caviae* species (GPIC^T^), usually positioned between the *C. felis* and *C. psittaci* clusters [2], [6], in the same group with LLG and POS *C. abortus* variant strains (Figure 1A, Figure S5A), even when filtering with Gblocks [70] was carried out. However, the ML tree (Figure 1A) indicated a relatively low BS support of 46%. Notably, a previous ML analysis recovered GPIC^T^ as a sister group to the *C. abortus* cluster [2], while in the present study exploratory Neighbor-Joining reconstruction (data not shown) led to a GPIC^T^ topology consistent with the currently accepted NJ-based phylogeny [2]. The position of GPIC^T^ will be discussed in more detail below.
- The same close evolutionary relationship between *C. psittaci* and *C. abortus* was recovered by ML and Bayesian analyses. *Chlamydophila abortus* subclusters branched off from a common ancestor with the *C. abortus* type strain B577^T^ and the *C. psittaci* variant strain Prk/Daruma, which, strikingly, shared identical 16S rRNA sequences (discussed below). The classical *C. abortus* strains were always grouped together in a subcluster (BS, 85%; PP, 1.00) and thereby separated from the LLG and POS *C. abortus* variant strains.

#### 23S domain I rRNA analysis

The trees derived from both phylogenetic approaches (Figure 1B, Figure S5B) showed overall agreement with previously published topologies based on full-length or 23S domain I rRNA alignments [2], [6], [66]. In the ML tree (Figure 1B), *C. psittaci* strains (6BC^T^, MN-VR122, and NJ1) grouped with *C. felis* and *C. caviae*, though with poor statistical support (BS 31%), were separated from the *C. psittaci* variant Prk/Daruma lineage. The latter, in both analyses, was the closest relative (BS, 88%; PP, 0.91) to the *C. abortus* cluster (BS, 70%; PP, 0.98), which branched further into the LLG/POS *C. abortus* (BS, 83%; PP, 0.95) and the classical *C. abortus* (BS, 86%; PP, 1.00) subclusters. Within the latter, strains FAG and VPG formed a distinct clade (BS, 68%; PP, 0.94).

#### 16S-23S IS analysis

The trees inferred from both phylogenetic approaches showed an almost identical overall topology (Figure 1C, Figure S5C), resembling a previously published 16S–23S IS tree [7]. The *C. psittaci* variant strain Prk/Daruma was again separated from the remaining *C. psittaci* strains. However, compared with the 16S and 23S trees, the evolutionary relationships of most *Chlamydophila* species were less well resolved. Nevertheless, a distinct *C. abortus* cluster is recovered again, with the LLG/POS *C. abortus* variant strains again forming a subcluster (BS, 65%; PP, 0.85).

### Phylogeny and definition of the *C. abortus* cluster and subclusters

The current rRNA-based phylogenetic analyses provided strong evidence that *C. abortus* has evolved from *C. psittaci*, which is in agreement with previous findings based on other gene analyses [6], [7]. However, the *C. abortus* cluster and subclusters should be verified and defined by analysis of derived (autapomorphic or apomorphic) characters in the rRNA molecules such as signature or unique nucleotides [69], [71]–[73]. A signature nucleotide in this context is a nucleotide residue found explicitly in a certain position within the sequences of the particular cluster or group, where the base that is present differs from those found in the majority of other bacteria. A nucleotide residue at a certain position is said to be unique when present in all strains of a particular group or cluster and absent, with no or only a few exceptions, in the strains of any other chlamydial group or cluster. The characterization of unique nucleotide features was restricted to the *Chlamydiales* (autapomorphic characters) or to the *Chlamydiaceae* or *Chlamydophila* (apomorphic characters) taxa.

#### 16S rRNA analysis

Among the five nucleotide differences observed between *C. abortus* and *C. psittaci* sequences (Table 2, Figure S2A and S2B), residue U277 represents a signature nucleotide for the classical *C. abortus* strains (see also Table 1). The subcluster of *C. abortus* classical strains is also supported by the residue A396 which is unique among all members of the order *Chlamydiales*. Perhaps the idiosyncratic U277 in the 16S molecule is the most pronounced attribute for classical *C. abortus*, since it corresponds to a highly conserved residue (discussed above). Interestingly, the *C. abortus* type strain B577^T^ does not share the U277 and A396 residues, presenting an identical sequence with the *C. psittaci* variant Prk/Daruma strain. It should be noted that a type strain is not necessarily the most representative member of a species [74]. On the other hand, 16S rRNA sequence identity could not be indicative of species identity in some cases [75]. The LLG and POS *C. abortus* strains do not share residues U277 and A396 either (Tables 1, 2), however, the corresponding subcluster could be strongly supported by the residue G681 which is unique among all members of the order *Chlamydiales* (Table 1; discussed above). The variable residue A80 could also be informative for the LLG/POS subcluster, however this represents an ancestral and shared (symplesiomorphic) character occurring in the *Simkaniaceae* sp. ancestor and shared by *C. caviae* (strain GPIC^T^) (see Table 1). At variable positions, identical residues are probably the result of multiple changes during the course of evolution, simulating an unchanged position (plesiomorphy). Such plesiomorphic-like sites may cause misleading branch attraction [20], like the one observed with *C. caviae* (strain GPIC^T^) resulting in its grouping with LLG and POS strains in the 16S rRNA tree (Figure 1A, Figure S5A). Other plesiomorphic characters shared by *C. abortus* and *C. caviae* at positions where nucleotide differences between *C. abortus* and *C. psittaci* occur (Table 2) could further intensify the branch attraction and affect the tree topology.

**Table 2:** Nucleotide differences between *C. abortus* and *C. psittaci* rRNA molecules compared with related taxa^a^.

Position in^b^	*C. abortus*	*C. psittaci*	*C. caviae*	*C. felis*	*C. pecorum*	*C. pneumoniae*	*C. trachomatis*	*C. muridarum*	*C. suis*	*Parachlamydiaceae*	Waddliaceae	*Simkaniaceae*	Domain *Bacteria* c
**16S rRNA**													
224	A	G/A*	A	G	A	G	A	A	A	A	A	G/A	A 33.01; G 14.17; U 41.17; C 11.57
**277**	**U**/C†	C	C	C	C	C	C	C	C	C	C	C	U 0.59; C 99.27; A 0.00; G 0.10
**396**	**A**/G†	G	G	G	G	G	G	G	G	G	G	G	A 37.42; G 30.29; C 31.05; U 1.20
1267	C	U/C*	C	U	U	U	C/U	C	U	U/C	C	A	C 66.45; U 30.80; A 0.73; G 0.26
1268	A	G/A*	A	G	G	G	G	G	G	G	G	A	A 12.57; G 85.42; C 0.17; U 0.09
**16S–23S Intergenic Spacer**													
49	A	G/A*	G	A	A	A	G/A	A	G/A	U	G	–	
55–56	–	U/–*	A	A	–	–	U	U	C	G	–	U	
185	C	A/C*	C	A	C	C	U	U	U	U	A	G	
192–193	–	A/–*	–	A	–	A	A	A	A	A	A	C	
198	A	U/A*	A	A	A	A	A	A	A	G	A	G	
**204**	**U**	C	C	C	C	C	C	C	C	U	–	C	
**23S rRNA (domain I)**													
**18**	**U**	C	C	C	C	C	C	C	C	C	C	C	U 18.73; C 80.88; A 0.40; G 0.00
132	A	G/A*	G	G	A	A	G	G	G	A	A	G/A	A 17.23; G 46.82; C 33.71; U 0.75
147	U	C/U*	C	C	U	U	C	C	C	U	U	C/U	U 37.78; C 25.19; G 34.44; A 1.11
**152**	**G**/A†	A	A	A	A	A	G	G	G	U/C	U	C/U	G 19.63; A 27.78; U 13.70; C 37.78
157	C	U/C*	C	C	C	U/C	C	C	C	C	C/U	C	C 62.22; U 11.11; G 15.93; A 2.96
**181–182**	**U**/C†	C	C	C	A	C	C	C	C	U/C	G	U/–	
240	G	A/G*	A	G	G	G	G	G	G	A/U	G	U	G 39.71; A 22.38; U 21.30; C 16.61
**297**	**C**	U/C*	U	U	U	U	U	U	U	C/A	C	A	C 23.38; U 29.86; A 25.90; G 20.50
**547**	C/**U** ‡	A/C*	C	C	C	C	A	A	A	U/A	A	U	C 16.13; U 20.79; A 53.76; G 9.32

a More than three accession numbers for each species or family, including the type strain, were analyzed (the majority of analyzed strains and their accession numbers are given in the Figures S2, S3, and S4).

b Nucleotide positions of the 16S and 23S domain I rRNA are given according to the *E. coli* J01695 secondary structure numbering system [35]; 16S–23S Intergenic Spacer positions based on primary structure is given according to the *C. abortus* type strain B577^T^ sequence (U68445). Signature and unique residues for *C. abortus* strains and the corresponding positions are shown in bold; see text for details. Dashes indicate nucleotide gaps in rRNA sequence comparison.

c Single base frequencies within domain *Bacteria* (dataset from http://www.rna.ccbb.utexas.edu/SAE/2A/nt_Frequency/).

† Nucleotide in *C. abortus* LLG/POS variant;

‡ Nucleotide in *C. abortus* FAG/VPG variant;

*Nucleotide in *C. psittaci* Prk/Daruma variant including the Prk/Daruma (acc. nos. D85710, AF481048), Prk46, Prk48, Prk49 (acc. nos. AB001809, AB001810, and AB001811, respectively), 84/2334 and 1V (acc. nos. AJ310736 and EF165622, respectively) strains.

#### 23S domain I rRNA analysis

Among the nine nucleotide differences observed between the *C. abortus* and *C. psittaci* sequences (Table 2, Figure S3A and S3B), residue U18 is unique for *C. abortus* strains, since it is not found in any other member of the order *Chlamydiales*, thereby defining the *C. abortus* cluster. Besides this, the residues in three other positions, namely 152, [181–182], and 297, could also be regarded as unique nucleotides. Residue C297, observed in all *C. abortus* strains but not among other *Chlamydiaceae* species, also could support the *C. abortus* cluster. Residues G152 and U[181–182] support the separation of the classical *C. abortus* subcluster, since they are not shared by other *Chlamydophila* or *Chlamydiaceae* species, respectively, as well as by the LLG/POS *C. abortus* variant (Tables 1, 2). The latter, LLG/POS variant subcluster, could be supported by the variable and informative residue G273 (Table 1). Finally, the group formed within *C. abortus* classical subcluster by the FAG and VPG strains is supported by the residue U547 which is not observed in other *Chlamydiaceae* members (Tables 1, 2).

#### 16S–23S IS analysis

Another unique character of *C. abortus* cluster, also detected by Van Loock et al. [7], is the residue U204 in the IS sequence (according to *C. abortus* B577^T^ sequence; acc. no. U68445) (Table 2). This residue is shared by both *C. abortus* subclusters (Table 2). Therefore, the LLG/POS *C. abortus* variant clade supported by U79 residue (Table 1) arises among other *C. abortus* strains (Figure 1C, Figure S5C). The topological difference of the “LLG/POS variant” in trees derived from the 16S–23S IS does not necessarily indicate a different path of evolution, since the IS region is more variable compared to 16S and 23S rRNAs.

Finally, as outlined in Table 2, at positions where differences between *C. abortus* and *C. psittaci* occur, the Prk/Daruma *C. psittaci* variant (including the Prk/Daruma, Prk46, Prk48, Prk49, 84/2334 and 1V avian strains) shared identical nucleotides with *C. abortus* strains. Nevertheless, this avian variant does not share the signature or unique *C. abortus* residues with only one exception, that of C297. The latter is particularly significant, since, based on the current rRNA phylogenetic analyses, the Prk/Daruma variant forms a distinct ancestral line for *C. abortus* supporting its intermediate position in the evolution of *C. abortus* from *C. psittaci* in agreement with previous reports [7], [61]. Recently, a multi-locus sequence typing scheme based on the partial sequences of seven housekeeping genes grouped the avian *C. psittaci* variant strain 84/2334 into *C. abortus* [76]. This does not contradict the above, as it is unlikely that independently evolving markers have preserved information on the same eras of evolutionary time [20].

### Remarks on the evolutionary relationships among *C. abortus* variants

Based on rRNA secondary structure sequence data, we have investigated the evolutionary relationships among known *Chlamydophila abortus* variant strains originated from a common geographical region. Our results suggest that *C. abortus* strains could be regarded as representing two distinct phylogenetic lineages designated “classical” and “LLG/POS variant”. On the basis of maximum likelihood and Bayesian phylogenetic analyses these lineages were reliably recovered as subclusters supported by the presence of derived characters, with the *C. abortus* type strain B577^T^ possibly representing an intermediate of the two lineages. The two *C. abortus* lineages, sharing three unique characters in the 23S domain I (residues U18 and C297) and 16S–23S IS (residue U204), but none in 16S (Table 2), could be distinguished on the basis of eight positions in the rRNA molecules (Tables 1, 2); four of these positions comprised nucleotides that appeared to be characteristic (signature or unique) of the “classical” lineage while three positions were unique for the “LLG/POS variant”. The U277 signature character, corresponding to a highly conserved residue of the 16S molecule, is the most pronounced attribute of the “classical” subcluster. Similarly, the unique G681 residue, conserved in a functionally strategic region also of the 16S molecule, is the most characteristic feature of the “LLG/POS variant”. Overall, the derived (signature or unique) *C. abortus* characters can serve as useful genetic markers for the identification of new strains before performing *C. abortus*-specific multilocus VNTR genotyping [17]. The rRNA-based phylogeny was consistent with the VNTR genotyping. In particular, the strains under investigation representing three different VNTR genotypes were also differentiated at least in one rRNA molecule (see also Table S2).

From an evolutionary perspective, both *C. abortus* lineages were found to be descendants of a common ancestor with the Prk/Daruma *C. psittaci* variant, and to have early diverged and separated during their evolution. Compared with the “classical” lineage, the “LLG/POS variant” has retained more ancestral features in the rRNA molecules as well as in other loci as gauged by the distinct similarity with *C. psittaci*-specific VNTR fragments [17]. The evolutionary events leading to rRNA sequence variations in both lineages have likely occurred once, as it is generally assumed for rRNA sequence evolution [20]. The observed rRNA sequence variations could possibly be explained by more rapid evolution due to a relatively recent shift to a host (ruminant), to which the *C. psittaci* variant ancestor had not been completely adapted. However, the FAG and VPG *C. abortus* strains, which represent the most common VNTR genotype of *C. abortus* in Greece and other countries [17], [77], have likely evolved from other “classical” strains, such as the FAS, following a gradual change (U547 residue) in the 23S domain I rRNA molecule.

Considering the relatively few sequence differences that lead to the classification of *Chlamydiaceae* into different species [2], [6] and based on the fact that most *C. abortus* derived characters corresponded to conserved residues, a subspecies status for each lineage may be applicable. The overall biological, biochemical and genotypic differentiation between LLG/POS variant and other *C. abortus* classical strains [13]–[18] in addition to their phylogenetic placement favor the delineation of these lineages as “subspecies”. Nevertheless, their systematics should be significantly aided by future sequence analyses of complete genomes [78].

In conclusion, the current rRNA secondary structure-based analysis and phylogenetic inference reveal new insights into how *C. abortus* variants of this study have differentiated during their evolution. The pattern and distribution of derived characters in functionally important regions of rRNA molecules could also make *C. abortus* a valuable model system for studies of molecular evolution in bacteria.

## Supporting Information

Figure S1 Schematic representation of the 16S, 16S-23S intergenic spacer (IS) and 23S domain I rDNA showing the four overlapping PCR-amplified rDNA fragments as well as the relative positions of the primers used. The positions (*^a^*) of the primers are given according to the sequences determined in this study (GenBank accession numbers EF486853-EF486857). Numbers in parentheses are positions of the 16S (*^b^*) and 23S domain I (*^c^*) rRNA genes according to *E. coli* numbering system.
(DOC)

Figure S2 16S rRNA secondary structure-based alignment of *Chlamydophila abortus* and other *Chlamydiales* sp. sequences (71 strains), created with the SINA Webaligner (SILVA SSU reference alignment [33]). Alignment segments corresponding to the structural elements bearing SNVs (helices H61, H240, H39, and H673 in which the LLG/POS variant presents SNVs at positions 80, 277, 396, and 681, respectively) are shown in **A**. The positions in which *C. abortus* and *C. psittaci* species present nucleotide differences (positions 224/H122, 277/H240, 396/H39, and 1267&1268/H1241) are shown in **A** and **B**. Helix numbering and nucleotide positions are according to the *E. coli* numbering system (Comparative RNA Web, CRW site [35]). Relevant positions are indicated in boldface and shaded with their paired base positions; the latter appear in normal font. Loops and bulges are indicated with grey letters. Alignments were used to generate the Tables 1 and 2 of the paper.
(DOC)

Figure S3 23S domain I rRNA secondary structure-based alignment of *Chlamydophila abortus* and other *Chlamydiales* sp. sequences (67 strains), created with the SINA Webaligner (SILVA LSU reference alignment [33]). The helices and multistem-loop in which the LLG/POS variant presents SNVs (H150, ML between H150 & H183, and H271, at positions 152, [181–182], and 273, respectively), as well as the hairpin-loop in which the FAG/VPG variant presents a SNV (HL of the H533 at position 547) are shown in **A**. The positions in which *C. abortus* and *C. psittaci* species present nucleotide differences (positions 18/H15, 132/H131, 147/H131, 152/H150, 157/H150, [181–182]/ML177–182, 240/H235, 297/H271, and 547/HL545–548) are shown in **A** and **B**. Helix numbering and nucleotide positions are according to the *E. coli* numbering system (Comparative RNA Web, CRW site [35]). Relevant positions are indicated in boldface and shaded with their paired base positions; the latter appear in normal font. Loops and bulges are indicated with grey letters. Alignments were used to generate the Tables 1 and 2 of the paper.
(DOC)

Figure S4 **A.** 16S-23S rRNA intergenic spacer (IS) multiple sequence alignment of *Chlamydophila abortus* and other *Chlamydiaceae* sp. (57 strains), generated with CLUSTAL X (1.83) [32]. The IS sequences alignment of *Parachlamydiaceae* sp., *Waddliaceae* sp. and *Simkaniaceae* sp. strains, generated manually on the basis of the *Chlamydiaceae* sp. consensus sequence, is shown under the latter (see also [66]). The position in which the LLG/POS variant presents a SNV (position 79), and the positions in which *C. abortus* and *C. psittaci* species (shaded by yellow color) present interspecies differences (positions 49, 55–56, 185, 192–193, 198 and 204) are shaded. Relevant positions are indicated based on the 222 bp sequence of the *C. abortus* type strain B577^T^ (U68445). Alignments were used to generate the Table 1 & 2 of the paper. **B.** Segment of stem region predicted to be formed between chlamydial 16S-23S rRNA IS and a complementary sequence of the 16S promoter [66]. The “-10 sequence” [68] is indicated with red letters.
(DOC)

Figure S5 Bayesian analysis (consensus trees) of 16S (**A**), 23S domain I (**B**), and 16S-23S IS (**C**) rRNA sequences of *C. abortus* strains and other *Chlamydiales* species. Numbers on branches indicate posterior probabilities. MrBayes version 3.1.2 was used [39]. The TreeGraph2 software [42] was used to display and manipulate the phylogenetic trees.
(DOC)

Table S1 Primer pairs and internal primer used for rDNA amplification and direct sequencing.
(DOC)

Table S2 Comparison of VNTR and rRNA genotypes.
(DOC)
