# Supplementary material for: Evolutionary Relationships among Chlamydophila abortus Variant Strains Inferred by rRNA Secondary Structure-Based Phylogeny
Source: PLoS One. 2011 May 24;6(5):e19813. doi: 10.1371/journal.pone.0019813 (PMC3101216; doi:10.1371/journal.pone.0019813)
Supplement: Figure S1 — Schematic representation of the 16S, 16S-23S intergenic spacer (IS) and 23S domain I rDNA showing the four overlapping PCR-amplified rDNA fragments as well as the relative positions of the primers used. The positions (a) of the primers are given according to the sequences determined in this study (GenBank accession numbers EF486853-EF486857). Numbers in parentheses are positions of the 16S (b) and 23S domain I (c) rRNA genes according to E. coli numbering system. (DOC) [file pone.0019813.s001.doc]

**Figure S1.**

**16S rRNA**

**23S domain I rRNA**

**16SFor**

**16SF**

**16SR**

**16SIGR**

**23R**

**23SIGR**

**16SF2**

**409Rev**

**Intergenic Spacer**

(*b*)

(*c*)

(*a*)

*^α^* Positions of the primers: **16SFor**, 1-20 (5-24)*^b^*; **16SIGR**, 329-313 (325-309)*^b^*; **16SF**, 38-56 (42-60)*^b^*; **16SR**, 1545-1528 (1539-1522)*^b^*; **16SF2**, 1403-1420 (1399-1416)*^b^*; **23R**, 1983-1965 (207-189)*^c^*;

**23SIGR**, >2396-2385 (582-562)*^c^*; **409Rev**, 1811-1792 (36-17)*^c^*.
