# Supplementary material for: Evolutionary Relationships among Chlamydophila abortus Variant Strains Inferred by rRNA Secondary Structure-Based Phylogeny
Source: PLoS One. 2011 May 24;6(5):e19813. doi: 10.1371/journal.pone.0019813 (PMC3101216; doi:10.1371/journal.pone.0019813)
Supplement: Figure S2 — 16S rRNA secondary structure-based alignment of Chlamydophila abortus and other Chlamydiales sp. sequences (71 strains), created with the SINA Webaligner (SILVA SSU reference alignment [33]). Alignment segments corresponding to the structural elements bearing SNVs (helices H61, H240, H39, and H673 in which the LLG/POS variant presents SNVs at positions 80, 277, 396, and 681, respectively) are shown in A. The positions in which C. abortus and C. psittaci species present nucleotide differences (positions 224/H122, 277/H240, 396/H39, and 1267&1268/H1241) are shown in A and B. Helix numbering and nucleotide positions are according to the E. coli numbering system (Comparative RNA Web, CRW site [35]). Relevant positions are indicated in boldface and shaded with their paired base positions; the latter appear in normal font. Loops and bulges are indicated with grey letters. Alignments were used to generate the Tables 1 and 2 of the paper. (DOC) [file pone.0019813.s002.doc]

**Figure S2 A.**

**H61*(61-82/87-106)**  **H240(240-259/267-286) H39(39-46/395-403)** **H673(673-690/697-717)**

**80** : 89 247 : **277**  45 : **396 681** **:** 709

D85709_B577 GUCGAACGGAA--UAA---UGACUUCGGUUG----UU-AUUUAGUGGC GAUAUCAGCUUGUUGGUGGGGUAAUGGCCUACCAAGGCUUUGACGUC GCGGCGUG CGACGCCGC GGAAUUCCACGUGUAG-CGGUGAA-AUGCGUAGAUAUGUGGAAGAAC

EF486856_LLG GUCGAACGGAA--UAA---UAACUUCGGUUG----UU-AUUUAGUGGC GAUAUCAGCUUGUUGGUGGGGUAAUGGCCUACCAAGGCUUUGACGUC GCGGCGUG CGACGCCGC GGAAUUCCGCGUGUAG-CGGUGAA-AUGCGUAGAUAUGUGGAAGAAC

EF486857_POS GUCGAACGGAA--UAA---UAACUUCGGUUG----UU-AUUUAGUGGC GAUAUCAGCUUGUUGGUGGGGUAAUGGCCUACCAAGGCUUUGACGUC GCGGCGUG CGACGCCGC GGAAUUCCGCGUGUAG-CGGUGAA-AUGCGUAGAUAUGUGGAAGAAC

EF486853_FAS GUCGAACGGAA--UAA---UGACUUCGGUUG----UU-AUUUAGUGGC GAUAUCAGCUUGUUGGUGGGGUAAUGGCCUACCAAGGUUUUGACGUC GCGGCGUG CAACGCCGC GGAAUUCCACGUGUAG-CGGUGAA-AUGCGUAGAUAUGUGGAAGAAC

EF486854_FAG GUCGAACGGAA--UAA---UGACUUCGGUUG----UU-AUUUAGUGGC GAUAUCAGCUUGUUGGUGGGGUAAUGGCCUACCAAGGUUUUGACGUC GCGGCGUG CAACGCCGC GGAAUUCCACGUGUAG-CGGUGAA-AUGCGUAGAUAUGUGGAAGAAC

EF486855_VPG GUCGAACGGAA--UAA---UGACUUCGGUUG----UU-AUUUAGUGGC GAUAUCAGCUUGUUGGUGGGGUAAUGGCCUACCAAGGUUUUGACGUC GCGGCGUG CAACGCCGC GGAAUUCCACGUGUAG-CGGUGAA-AUGCGUAGAUAUGUGGAAGAAC

CR848038_S26/3 GUCGAACGGAA--UAA---UGACUUCGGUUG----UU-AUUUAGUGGC GAUAUCAGCUUGUUGGUGGGGUAAUGGCCUACCAAGGUUUUGACGUC GCGGCGUG CAACGCCGC GGAAUUCCACGUGUAG-CGGUGAA-AUGCGUAGAUAUGUGGAAGAAC

U76710_EBA GUCGAACGGAA--UAA---UGACUUCGGUUG----UU-AUUUAGUGGC GAUAUCAGCUUGUUGGUGGGGUAAUGGCCUACCAAGGUUUUGACGUC GCGGCGUG CAACGCCGC GGAAUUCCACGUGUAG-CGGUGAA-AUGCGUAGAUAUGUGGAAGAAC

Z49871_EAE GUCGAACGGAA--UAA---UGACUUCGGUUG----UU-AUUUAGUGGC GAUAUCAGCUUGUUGGUGGGGUAAUGGCCUACCAAGGUUUUGACGUC GCGGCGUG CAACGCCGC GGAAUUCCACGUGUAG-CGGUGAA-AUGCGUAGAUAUGUGGAAGAAC

Z49872_OEA GUCGAACGGAA--UAA---UGACUUCGGUUG----UU-AUUUAGUGGC GAUAUCAGCUUGUUGGUGGGGUAAUGGCCUACCAAGGUUUUGACGUC GCGGCGUG CAACGCCGC GGAAUUCCACGUGUAG-CGGUGAA-AUGCGUAGAUAUGUGGAAGAAC

U68447_6BC GUCGAACGGAA--UAA---UGACUUCGGUUG----UU-AUUUAGUGGC GAUAUCAGCUUGUUGGUGGGGUAAUGGCCUACCAAGGCUUUGACGUC GCGGCGUG CGACGCCGC GGAAUUCCACGUGUAG-CGGUGAA-AUGCGUAGAUAUGUGGAAGAAC

U68419_NJ1 GUCGAACGGAA--UAA---UGACUUCGGUUA----UU-AUUUAGUGGC GAUAUCAGCUUGUUGGUGGGGUAACGGCCUACCAAGGCUUUGACGUC GCGGCGUG CGACGCCGC GGAAUUCCACGUGUAG-CGGUGAA-AUGCGUAGAUAUGUGGAAGAAC

D85713_Prt/GCP-1 GUCGAACGGAA--UAA---UGACUUCGGUUG----UU-AUUUAGUGGC GAUAUCAGCUUGUUGGUGGGGUAAUGGCCUACCAAGGCUUUGACGUC GCGGCGUG CGACGCCGC GGAAUUCCACGUGUAG-CGGUGAA-AUGCGUAGAUAUGUGGAAGAAC

D85712_Frt-Hu/Ca110 GUCGAACGGAA--UAA---UGACUUCGGUUG----UU-AUUUAGUGGC GAUAUCAGCUUGUUGGUGGGGUAAUGGCCUACCAAGGCUUUGACGUC GCGGCGUG CGACGCCGC GGAAUUCCACGUGUAG-CGGUGAA-AUGCGUAGAUAUGUGGAAGAAC

D85711_Hu/Borg GACGAACGGAA--UAA---UGACUCCGGUUA----UU-AUUUAGUGGC GAUAUCAGCUUGUUGGUGGGGUAACGGCCUACCAAGGCUUUGACGUC GCGGCGUG CGACGCCGC GGAAUUCCACGUGUAG-CGGUGAA-AUGCGUAGAUAUGUGGAAGAAC

D85710_Prk/Daruma GUCGAACGGAA--UAA---UGACUUCGGUUG----UU-AUUUAGUGGC GAUAUCAGCUUGUUGGUGGGGUAAUGGCCUACCAAGGCUUUGACGUC GCGGCGUG CGACGCCGC GGAAUUCCACGUGUAG-CGGUGAA-AUGCGUAGAUAUGUGGAAGAAC

AB001809_Prk46 GUCGAACGGAA--UAA---UGACUUCGGUUG----UU-AUUUAGUGGC GAUAUCAGCUUGUUGGUGGGGUAAUGGCCUACCAAGGCUUUGACGUC GCGGCGUG CGACGCCGC GGAAUUCCACGUGUAG-CGGUGAA-AUGCGUAGAUAUGUGGAAGAAC

AB001810_Prk48 GUCGAACGGAA--UAA---UGACUUCGGUUG----UU-AUUUAGUGGC GAUAUCAGCUUGUUGGUGGGGUAAUGGCCUACCAAGGCUUUGACGUC GCGGCGUG CGACGCCGC GGAAUUCCACGUGUAG-CGGUGAA-AUGCGUAGAUAUGUGGAAGAAC

AB001811_Prk49 GUCGAACGGAA--UAA---UGACUUCGGUUG----UU-AUUUAGUGGC GAUAUCAGCUUGUUGGUGGGGUAAUGGCCUACCAAGGCUUUGACGUC GCGGCGUG CGACGCCGC GGAAUUCCACGUGUAG-CGGUGAA-AUGCGUAGAUAUGUGGAAGAAC

AB001779_Bud1 GUCGAACGGAA--UAA---UGACUUCGGUUG----UU-AUUUAGUGGC GAUAUCAGCUUGUUGGUGGGGUAAUGGCCUACCAAGGCUUUGACGUC GCGGCGUG CGACGCCGC GGAAUUCCGCGUGUAG-CGGUGAA-AUGCGUAGAUAUGUGGAAGAAC

AB001780_Bud11F GUCGAACGGAA--UAA---UGACUUCGGUUG----UU-AUUUAGUGGC GAUAUCAGCUUGUUGGUGGGGUAAUGGCCUACCAAGGCUUUGACGUC GCGGCGUG CGACGCCGC GGAAUUCCACGUGUAG-CGGUGAA-AUGCGUAGAUAUGUGGAAGAAC

AB001782_Bud5695 GUCGAACGGAA--UAA---UGACUUCGGUUG----UU-AUUUAGUGGC GAUAUCAGCUUGUUGGUGGGGUAAUGGCCUACCAAGGCUUUGACGUC GCGGCGUG CGACGCCGC GGAAUUCCACGUGUAG-CGGUGAA-AUGCGUAGAUAUGUGGAAGAAC

AB001792_P1015 GUCGAACGGAA--UAA---UGACUUCGGUUG----UU-AUUUAGUGGC GAUAUCAGCUUGUUGGUGGGGUAAUGGCCUACCAAGGCUUUGACGUC GCGGCGUG CGACGCCGC GGAAUUCCACGUGUAG-CGGUGAA-AUGCGUAGAUAUGUGGAAGAAC

AB001808_PCM9 GUCGAACGGAA--UAA---UGACUUCGGUUG----UU-AUUUAGUGGC GAUAUCAGCUUGUUGGUGGGGUAAUGGCCUACCAAGGCUUUGACGUC GCGGCGUG CGACGCCGC GGAAUUCCAUGUGUAG-CGGUGAA-AUGCGUAGAUAUGUGGAAGAAC

AB001807_PCM55 GUCGAACGGAA--UAA---UGACUUCGGUUG----UU-AUUUAGUGGC GAUAUCAGCUUGUUGGUGGGGUAAUGGCCUACCAAGGCUUUGACGUC GCGGCGUG CGACGCCGC GGAAUUCCAUGUGUAG-CGGUGAA-AUGCGUAGAUAUGUGGAAGAAC

AB001806_PCM44 GUCGAACGGAA--UAA---UGACUUCGGUUG----UU-AUUUAGUGGC GAUAUCAGCUUGUUGGUGGGGUAAUGGCCUACCAAGGCUUUGACGUC GCGGCGUG CGACGCCGC GGAAUUCCAUGUGUAG-CGGUGAA-AUGCGUAGAUAUGUGGAAGAAC

AB001804_PCM27 GUCGAACGGAA--UAA---UGACUUCGGUUG----UU-AUUUAGUGGC GAUAUCAGCUUGUUGGUGGGGUAAUGGCCUACCAAGGCUUUGACGUC GCGGCGUG CGACGCCGC GGAAUUCCACGUGUAG-CGGUGAA-AUGCGUAGAUAUGUGGAAGAAC

AB001802_PgAu46 GUCGAACGGAA--UAA---UGACUUCGGUUA----UU-AUUUAGUGGC GAUAUCAGCUUGUUGGUGGGGUAACGGCCUACCAAGGCUUUGACGUC GCGGCGUG CGACGCCGC GGAAUUCCACGUGUAG-CGGUGAA-AUGCGUAGAUAUGUGGAAGAAC

AB001795_P1307 GUCGAACGGAA--UAA---UGACUUCGGUUG----UU-AUUUAGUGGC GAUAUCAGCUUGUUGGUGGGGUAAUGGCCUACCAAGGCUUUGACGUC GCGGCGUG CGACGCCGC GGAAUUCCACGUGUAG-CGGUGAA-AUGCGUAGAUAUGUGGAAGAAC

AB001814_T3 GUCGAACGGAA--UAA---UGACUUCGGUUA----UU-AUUUAGUGGC GAUAUCAGCUUGUUGGUGGGGUAACGGCCUACCAAGGCUUUGACGUC GCGGCGUG CGACGCCGC GGAAUUCCACGUGUAG-CGGUGAA-AUGCGUAGAUAUGUGGAAGAAC

AB001815_T4 GUCGAACGGAA--UAA---UGACUUCGGUUG----UU-AUUUAGUGGC GAUAUCAGCUUGUUGGUGGGGUAAUGGCCUACCAAGGCUUUGACGUC GCGGCGUG CGACGCCGC GGAAUUCCACGUGUAG-CGGUGAA-AUGCGUAGAUAUGUGGAAGAAC

AB001791_Ohmiya GUCGAACGGAA--UAA---UGACUUCGGUUG----UU-AUUUAGUGGC GAUAUCAGCUUGUUGGUGGGGUAAUGGCCUACCAAGGCUUUGACGUC GCGGCGUG CGACGCCGC GGAAUUCCACGUGUAG-CGGUGAA-AUGCGUAGAUAUGUGGAAGAAC

AB001787_Itoh GUCGAACGGAA--UAA---UGACUUCGGUUG----UU-AUUUAGUGGC GAUAUCAGCUUGUUGGUGGGGUAAUGGCCUACCAAGGCUUUGACGUC GCGGCGUG CGACGCCGC GGAAUUCCACGUGUAG-CGGUGAA-AUGCGUAGAUAUGUGGAAGAAC

AB285329_CPX0308 GUCGAACGGAA--UAA---UGACUUCGGUUA----UU-AUUUAGUGGC GAUAUCAGCUUGUUGGUGGGGUAAUGGCCUACCAAGGCUUUGACGUC GCGGCGUG CGACGCCGC GGAAUUCCACGUGUAG-CGGUGAA-AUGCGUAGAUAUGUGGAAGAAC

D85708_GPIC GUCAAACGGAA--UAA---UAGCGUCGGGUA----UU-AUUUAGUGGC GAUAUCAGCUUGUUGGUGGGGUAAUGGCCUACCAAGGCUUUGACGUC GCGGCGUG CGACGCCGC GGAAUUCCACGUGUAG-CGGUGAA-AUGCGUAGAUAUGUGGAAGAAC

AE015925_GPIC GUCGAACGGAA--UAA---UAGCUUCGGUUA----UU-AUUUAGUGGC GAUAUCAGCUUGUUGGUGGGGUAAUGGCCUACCAAGGCUUUGACGUC GCGGCGUG CGACGCCGC GGAAUUCCACGUGUAG-CGGUGAA-AUGCGUAGAUAUGUGGAAGAAC

D85701_Fe_Baker GUCGAACGGAA--CAA---UGACUUCGGUUG----UU-GUUUAGUGGC GAUAUCAGCUUGUUGGUGGGGUAAUGGCCUACCAAGGCUUUGACGUC GCGGCGUG CGACGCCGC GGAAUUCCACGUGUAG-CGGUGAA-AUGCGUAGAUAUGUGGAAGAAC

D85706_Fe_Cello GUCGAACGGAA--CAA---UGACUUCGGUUG----UU-GUUUAGUGGC GAUAUCAGCUUGUUGGUGGGGUAAUGGCCUACCAAGGCUUUGACGUC GCGGCGUG CGACGCCGC GGAAUUCCACGUGUAG-CGGUGAA-AUGCGUAGAUAUGUGGAAGAAC

D85702_Fe/145 GUCGAACGGAA--CAA---UGACUUCGGUUG----UU-GUUUAGUGGC GAUAUCAGCUUGUUGGUGGGGUAAUGGCCUACCAAGGCUUUGACGUC GCGGCGUG CGACGCCGC GGAAUUCCACGUGUAG-CGGUGAA-AUGCGUAGAUAUGUGGAAGAAC

AP006861_Fe/C-56 GUCGAACGGAA--CAA---UGACUUCGGUUG----UU-GUUUAGUGGC GAUAUCAGCUUGUUGGUGGGGUAAUGGCCUACCAAGGCUUUGACGUC GCGGCGUG CGACGCCGC GGAAUUCCACGUGUAG-CGGUGAA-AUGCGUGGAUAUGUGGAAGAAC

D88317_E58 GUCGAACGGAA--UAA---UGGCUUCGGCUA----UU-AUUUAGUGGC GAUAUCAGCUUGUUGGUGGGGUAAUGGCCUACCAAGGCUAUGACGUC GCGGCGUG CGACGCCGC GGAAUUCCACGUGUAG-CGGUGAA-AUGCGUAGAUAUGUGGAAGAAC

U73785_Bo/E8 ----------------------------------------UUAGUGGC GAUAUCAGCUUGUUGGUGGGGUAAUGGCCUACCAAGGCUAUGACGUC -------- CGACGCCGC GGAAUUCCACGUGUAG-CGGUGAA-AUGCGUAGAUAUGUGGAAGAAC

U73782_BE ----------------------------------------UUAGUGGC GAUAUCAGCUUGUUGGUGGGGUAAUGGCCUACCAAGGCUAUGACGUC -------- CGACGCCGC GGAAUUCCACGUGUAG-CGGUGAA-AUGCGUAGAUAUGUGGAAGAAC

D85716_Ov/IPA GUCGAACGGAA--UAA---UGGCUUCGGCUA----UU-AUUUAGUGGC GAUAUCAGCUUGUUGGUGGGGUAAUGGCCUACCAAGGCUAUGACGUC GCGGCGUG CGACGCCGC GGAAUUCCACGUGUAG-CGGUGAA-AUGCGUAGAUAUGUGGAAGAAC

D85715_Maeda GUCGAACGGAA--UAA---UGGCUCCGGCUA----UU-AUUUAGUGGC GAUAUCAGCUUGUUGGUGGGGUAAUGGCCUACCAAGGCUAUGACGUC GCGGCGUG CGACGCCGC GGAAUUCCACGUGUAG-CGGUGAA-AUGCGUAGAUAUGUGGAAGAAC

D85714_Shizuoka GUCGAACGGAA--UAA---UGGCUUCGGCUA----UU-AUUUAGUGGC GAUAUCAGCUUGUUGGUGGGGUAAUGGCCUACCAAGGCUAUGACGUC GCGGCGUG CGACGCCGC GGAAUUCCACGUGUAG-CGGUGAA-AUGCGUAGAUAUGUGGAAGAAC

D85717_Koala_II GUCGAACGGAA--UAA---UGGCUUCGGCUA----UU-AUUUAGUGGC GAUAUCAGCUUGUUGGUGGGGUAAUGGCCUACCAAGGCUAUGACGUC GCGGCGUG CGACGCCGC GGAAUUCCACGUGUAG-CGGUGAA-AUGCGUAGAUAUGUGGAAGAAC

L06108_TW-183 GUCGAACGGAA--UAA---UGACUUCGGUUG----UU-AUUUAGUGGC GAUAUCAGCUUGUUGGUGGGGUAAAAGCCCACCAAGGCGAUGACGUC GCGGCGUG CGACGCCGC GGAAUUCCACGUGUAG-CGGUGAA-AUGCGUAGAUAUGUGGAAGAAC

AE001363_CWL029 GUCGAACGGAA--UAA---UGACUUCGGUUG----UU-AUUUAGUGGC GAUAUCAGCUUGUUGGUGGGGUAAAAGCCCACCAAGGCGAUGACGUC GCGGCGUG CGACGCCGC GGAAUUCCACGUGUAG-CGGUGAA-AUGCGUAGAUAUGUGGAAGAAC

DQ444323_WBB GUCGAACGGAA--UAA---UGACUUCGGUUG----UU-AUUUAGUGGC GAUAUCAGCUUGUUGGUGGGGUAAAAGCCCACCAAGGCGAUGACGUC GCGGCGUG CGACGCCGC GGAAUUCCACGUGUAG-CGGUGAA-AUGCGUAGAUAUGUGGAAGAAC

AF139200_CPXT1 GUCGAACGGAA--UAA---UAAAUUCGGUUG----UU-AUUUAGUGGC GAUAUCAGCUUGUUGGUGGGGUAAAAGCCCACCAAGGCGAUGACGUC GCGGCGUG CGACGCCGC GGAAUUCCACGUGUAG-CGGUGAA-AUGCGUAGAUAUGUGGAAGAAC

U68426_N16 GUCGAACGGAA--UAA---UGACUUCGGUUG----UU-GUUUAGUGGC GAUAUCAGCUUGUUGGUGGGGUAAAAGCCCACCAAGGCGAUGACGUC ---GCGUG CGACGCCGC GGAAUUCCACGUGUAG-CGGUGAA-AUGCGUAGAUAUGUGGAAGAAC

FJ236984_LPCoLN GUCGAACGGAA--UAA---UGACUUCGGUUG----UU-AUUUAGUGGC GAUAUCAGCUUGUUGGUGGGGUAAAAGCCCACCAAGGCGAUGACGUC GCGGCGUG CGACGCCGC GGAAUUCCACGUGUAG-CGGUGAA-AUGCGUAGAUAUGUGGAAGAAC

D89067_A/HAR-13 GUCGAACGGAG--CAA---UUGUUUCGACGA----UU-GUUUAGUGGC GAUAUCAGCUAGUUGGUGGGGUAAAGGCCUACCAAGGCUAUGACGUC GCGGCGUG CGACGCCGC GGAAUUUCACGUGUAG-CGGUGAA-AUGCGUAGAUAUGUGGAAGAAC

D85719__B/TW-5/OT GUCGAACGGAG--CAA---UUGUUUCGACGA----UU-GUUUAGUGGC GAUAUCAGCUAGUUGGUGGGGUAAAGGCCUACCAAGGCUAUGACGUC GCGGCGUG CGACGCCGC GGAAUUUCACGUGUAG-CGGUGAA-AUGCGUAGAUAUGUGGAAGAAC

D85720_C/TW-3/OT GUCGAACGGAG--CAA---UUGUUUCGACGA----UU-GUUUAGUGGC GAUAUCAGCUAGUUGGUGGGGUAAAGGCCUACCAAGGCUAUGACGUC GCGGCGUG CGACGCCGC GGAAUUUCACGUGUAG-CGGUGAA-AUGCGUAGAUAUGUGGAAGAAC

D85721_D/TW-3/CX GUCGAACGGAG--CAA---UUGUUUCGACGA----UU-GUUUAGUGGC GAUAUCAGCUAGUUGGUGGGGUAAAGGCCUACCAAGGCUAUGACGUC GCGGCGUG CGACGCCGC GGAAUUUCACGUGUAG-CGGUGAA-AUGCGUAGAUAUGUGGAAGAAC

AE001273_D/UW-3/CX GUCGAACGGAG--CAA---UUGUUUCGACGA----UU-GUUUAGUGGC GAUAUCAGCUAGUUGGUGGGGUAAAGGCCUACCAAGGCUAUGACGUC GCGGCGUG CGACGCCGC GGAAUUUCACGUGUAG-CGGUGAA-AUGCGUAGAUAUGUGGAAGAAC

D85722_E/UW-5/CX GUCGAACGGAG--CAA---UUGUUUCGGCAA----UU-GUUUAGUGGC GAUAUCAGCUAGUUGGUGGGGUAAAGGCCUACCAAGGCUAUGACGUC GCGGCGUG CGACGCCGC GGAAUUUCACGUGUAG-CGGUGAA-AUGCGUAGAUAUGUGGAAGAAC

DQ019301_F/IC/CAL3 GUCGAACGGAG--CAA---UUGUUUCGGCAA----UU-GUUUAGUGGC GAUAUCAGCUAGUUGGUGGGGUAAAGGCCUACCAAGGCUAUGACGUC GCGGCGUG CGACGCCGC GGAAUUUCACGUGUAG-CGGUGAA-AUGCGUAGAUAUGUGGAAGAAC

U68443_L2/434/BU GUCGAACGGAG--CAA---UUGUUUCGGCAA----UU-GUUUAGUGGC GAUAUCAGCUAGUUGGUGGGGUAAAGGCCUACCAAGGCUAUGACGUC GCGGCGUG CGACGCCGC GGAAUUUCACGUGUAG-CGGUGAA-AUGCGUAGAUAUGUGGAAGAAC

D85718_MoPn GUCGAACGGAA--CAA---UUGCUUCGGUGA----UU-GUUUAGUGGC GAUAUCAGCUAGUUGGUGGGGUAAAGGCCUACCAAGGCUAUGACGUC GCGGCGUG CGACGCCGC GGAAUUUCACGUGUAG-CGGUGAA-AUGCGUAGAUAUGUGGAAGAAC

U68437_SFPD GUCGAACGGAA--CAA---UUGCUUCGGUGA----UU-GUUUAGUGGC GAUAUCAGCUAGUUGGUGGGGUAAAGGCCUACCAAGGCUAUGACGUC GCGGCGUG CGACGCCGC GGAAUUUCACGUGUAG-CGGUGAA-AUGCGUAGAUAUGUGGAAGAAC

U73110_S45 GUCGAACGGAA--CGG---UU--UUAU--AG----CU-GUUUAGUGGC GAUAUCAGCUAGUUGGUGGGGUAAAGGCCUACCAAGGCGAUGACGUC GCGGCGUG CGACGCCGC GGAAUUUCACGUGUAG-CGGUGAA-AUGCGUAGAUAUGUGGAAGAAC

U68420_R22 GUCGAACGGAA--CAG---UU--UUU---AA----CU-GUUUAGUGGC GAUAUCAGCUAGUUGGUAGGGUAAAGGCCUACCAAGGCUAUGACGUC GCGGCGUG CGACGCCGC GGAAUUUCACGUGUAG-CGGUGAA-AUGCGUAGAUAUGUGGAAGAAC

Y07556_Bn9 GUCGAACGAAA--CGG-------GCAA--------CC-GUUUAGUGGC GAUAUCAGCUAGUUGGUGUGGUAAUGGCGCACCAAGGCUAAGACGUC ACGGCGUG CGACGCCGU GGAAUUCCACAUGUAG-CGGUGAA-AUGCGUAGAUAUGUGGAAGAAC

DQ309029_Seine GUCGAACGAAA--CGG-------GCAA--------CC-GUUUAGUGGC GAUAUCAGCUAGUUGGUGUGGUAAUGGCGCACCAAGGCUAAGACGUC -CGGCGUG CGACGCCGU GGAAUUCCACAUGUAG-CGGUGAA-AUGCGUAGAUAUGUGGAAGAAC

AF042496_WSU_86-1044 GUCGAACGAAG--UGU----GCUCUUGAGU-----GCAACUUAGUGGC GAUAUCAGCUAGUUGGUGAAGUAAAGGCUCACCAAGGCUAAGACGUC ACGGCGUG CGACGCCGU GGAAUUCCCCAUGUUGCCGGUGAAAAUGCGUAGAUAUUUGGAAAAAC

AF346001_2032/99 GUCGAACGAAG--UGU----GCUCUUGAGU-----GC-ACUUAGUGGC GAUAUCAGCUAGUUGGUGAGGUAAAGGCUCACCAAGGCUAAGACGUC -CGGCGUG CGACGCCGU GGAAUUCCACAUGUAG-CGGUGAA-AUGCGUAGAUAUGUGGAAGAAC

U68460_Z GUCGAACGAAG--UAG---UAA-CUUG-UUA----CU-ACUUAGUGGC GAUAUCAGCUUGUUGGUGUGGUAAAGGCGCACCAAGGCUAAGACGUC ACGGCGUG CGACGCCGU GGAAUUCCACGUGUAG-CGGUGAA-AUGCGUAGAUAUGUAGAAGAAC

AY140910_YaeL GUCGAACGCAG--UAG-------CUUG--------CU-ACUGAGUGGC GAUAUCAGCUUGUUGGUGUGGUAAUGGCGCACCAAGGCUAAGACGUC ACGGCGUG CGACGCCGU GGAAUUCCAAGUGUAG-CGGUGAA-AUGCGUAGAUAUUUGGAAGAAC

J01695_E._coli GUCGAACG-GUAACAGGAAGAAGCUUGCUUCUUUGCU-GACGAGUGGC GGGAUUAGCUAGUAGGUGGGGUAACGGCUCACCUAGGCGACGAUCCC GCGGCAGG CCAUGCCGC AGAAUUCCAGGUGUAG-CGGUGAA-AUGCGUAGAGAUCUGGAGGAAU

*For H61 see also: Woese, Winker, Gutell. 1990. Architecture of ribosomal RNA: constraints on the sequence of "tetra-loops". Proc Natl Acad Sci USA.87,8467–8471;

**Ossewaarde and Meijer.** 1999. Molecular evidence for the existence of additional members of the order Chlamydiales. Microbiology 145,411-417)

**Figure S2 B.**

**H122(122-142/221-239) H1241(1241-1265/1270-1296)**

139 : **224 1267/1268**

D85709_B577 ACAUAGAUAAUCUGUCCUCAAC GUUAAGGGAGAGUCUAUGG GGCCAGUACAGAAGGUAGCAAUAUCGCAAGAUGGAGCAAAUCCUC-AAAGCUGGCC

EF486856_LLG ACAUAGAUAAUCUGUCCUCAAC GUUAAGGGAGAGUCUAUGG GGCCAGUACAGAAGGUAGCAAUAUCGCAAGAUGGAGCAAAUCCUC-AAAGCUGGCC

EF486857_POS ACAUAGAUAAUCUGUCCUCAAC GUUAAGGGAGAGUCUAUGG GGCCAGUACAGAAGGUAGCAAUAUCGCAAGAUGGAGCAAAUCCUC-AAAGCUGGCC

EF486853_FAS ACAUAGAUAAUCUGUCCUCAAC GUUAAGGGAGAGUCUAUGG GGCCAGUACAGAAGGUAGCAAUAUCGCAAGAUGGAGCAAAUCCUC-AAAGCUGGCC

EF486854_FAG ACAUAGAUAAUCUGUCCUCAAC GUUAAGGGAGAGUCUAUGG GGCCAGUACAGAAGGUAGCAAUAUCGCAAGAUGGAGCAAAUCCUC-AAAGCUGGCC

EF486855_VPG ACAUAGAUAAUCUGUCCUCAAC GUUAAGGGAGAGUCUAUGG GGCCAGUACAGAAGGUAGCAAUAUCGCAAGAUGGAGCAAAUCCUC-AAAGCUGGCC

CR848038_S26/3 ACAUAGAUAAUCUGUCCUCAAC GUUAAGGGAGAGUCUAUGG GGCCAGUACAGAAGGUAGCAAUAUCGCAAGAUGGAGCAAAUCCUC-AAAGCUGGCC

U76710_EBA ACAUAGAUAAUCUGUCCUCAAC GUUAAGGGAGAGUCUAUGG GGCCAGUACAGAAGGUAGCAAUAUCGCAAGAUGGAGCAAAUCCUC-AAAGCUGGCC

Z49871_EAE ACAUAGAUAAUCUGUCCUCAAC GUUAAGGGAGAGUCUAUGG GGCCAGUACAGAAGGUAGCAAUAUCGCAAGAUGGAGCAAAUCCUC-AAAGCUGGCC

Z49872_OEA ACAUAGAUAAUCUGUCCUCAAC GUUAAGGGAGAGUCUAUGG GGCCAGUACAGAAGGUAGCAAUAUCGCAAGAUGGAGCAAAUCCUC-AAAGCUGGCC

U68447_6BC ACAUAGAUAAUCUGUCCUCAAC GUUGAGGGAGAGUCUAUGG GGCCAGUACAGAAGGUAGCAAUAUCGUGAGAUGGAGCAAAUCCUC-AAAGCUGGCC

U68419_NJ1 ACAUAGAUAAUCUGUCCUCAAC GUUGAGGGAGAGUCUAUGG GGCCAGUACAGAAGGUAGCAAUAUCGUGAGAUGGAGCAAAUCCUC-AAAGCUGGCC

D85713_Prt/GCP-1 ACAUAGAUAAUCUGUCCUCAAC GUUGAGGGAGAGUCUAUGG GGCUAGUACAGAAGGUAGCAAUAUCGUGAGAUGGAGCAAAUCCUC-AAAGCUAGCC

D85712_Frt-Hu/Ca110 ACAUAGAUAAUCUGUCCUCAAC GUUGAGGGAGAGUCUAUGG GGCCAGUACAGAAGGUAGCAAUAUCGUGAGAUGGAGCAAAUCCUC-AAAGCUGGCC

D85711_Hu/Borg ACAUAGAUAAUCUGUCCUCAAC GUUGAGGGAGAGUCUAUGG GGCCAGUACAGAAGGUAGCAAUAUCGUGAGAUGGAGCAAAUCCUC-AAAGCUGGCC

D85710_Prk/Daruma ACAUAGAUAAUCUGUCCUCAAC GUUAAGGGAGAGUCUAUGG GGCCAGUACAGAAGGUAGCAAUAUCGCAAGAUGGAGCAAAUCCUC-AAAGCUGGCC

AB001809_Prk46 ACAUAGAUAAUCUGUCCUCAAC GUUAAGGGAGAGUCUAUGG GGCCAGUACAGAAGGUAGCAAUAUCGCAAGAUGGAGCAAAUCCUC-AAAGCUGGCC

AB001810_Prk48 ACAUAGAUAAUCUGUCCUCAAC GUUAAGGGAGAGUCUAUGG GGCCAGUACAGAAGGUAGCAAUAUCGCAAGAUGGAGCAAAUCCUC-AAAGCUGGCC

AB001811_Prk49 ACAUAGAUAAUCUGUCCUCAAC GUUAAGGGAGAGUCUAUGG GGCCAGUACAGAAGGUAGCAAUAUCGCAAGAUGGAGCAAAUCCUC-AAAGCUGGCC

AB001779_Bud1 ACAUAGAUAAUCUGUCCUCAAC GUUGAGGGAGAGUCUAUGG GGCCAGUACAGAAGGUAGCAAUAUCGUGAGAUGGAGCAAAUCCUC-AAAGCUGGCC

AB001780_Bud11F ACAUAGAUAAUCUGUCCUCAAC GUUGAGGGAGAGUCUAUGG GGCCAGUACAGAAGGUAGCAAUAUCGUGAGAUGGAGCAAAUCCUC-AAAGCUGGCC

AB001782_Bud5695 ACAUAGAUAAUCUGUCCUCAAC GUUGAGGGAGAGUCUAUGG GGCCAGUACAGAAGGUAGCAAUAUCGUGAGAUGGAGCAAAUCCUC-AAAGCUGGCC

AB001792_P1015 ACAUAGAUAAUCUGUCCUCAAC GUUGAGGGAGAGUCUAUGG GGCCAGUACAGAAGGUAGCAAUAUCGUGAGAUGGAGCAAAUCCUC-AAAGCUGGCC

AB001808_PCM9 ACAUAGAUAAUCUGUCCUCAAC GUUGAGGGAGAGUCUAUGG GGCCAGUACAGAAGGUAGCAAUAUCGUGAGAUGGAGCAAAUCCUC-AAAGCUGGCC

AB001807_PCM55 ACAUAGAUAAUCUGUCCUCAAC GUUGAGGGAGAGUCUAUGG GGCCAGUACAGAAGGUAGCAAUAUCGUGAGAUGGAGCAAAUCCUC-AAAGCUGGCC

AB001806_PCM44 ACAUAGAUAAUCUGUCCUCAAC GUUGAGGGAGAGUCUAUGG GGCCAGUACAGAAGGUAGCAAUAUCGUGAGAUGGAGCAAAUCCUC-AAAGCUGGCC

AB001804_PCM27 ACAUAGAUAAUCUGUCCUCAAC GUUGAGGGAGAGUCUAUGG GGCCGGUACAGAAGGUAGCAAUAUCGUGAGAUGGAGCAAAUCCUC-AAAGCUGGCC

AB001802_PgAu46 ACAUAGAUAAUCUGUCCUCAAC GUUGAGGGAGAGUCUAUGG GGCCAGUACAGAAGGUAGCAAUAUCGUGAGAUGGAGCAAAUCCUC-AAAGCUGGCC

AB001795_P1307 ACAUAGAUAAUCUGUCCUCAAC GUUGAGGGAGAGUCUAUGG GGCCGGUACAGAAGGUAGCAAUAUCGUGAGAUGGAGCAAAUCCUC-AAAGCUGGCC

AB001814_T3 ACAUAGAUAAUCUGUCCUCAAC GUUGAGGGAGAGUCUAUGG GGCCAGUACAGAAGGUAGCAAUAUCGUGAGAUGGAGCAAAUCCUC-AAAGCUGGCC

AB001815_T4 ACAUAGAUAAUCUGUCCUCAAC GUUGAGGGAGAGUCUAUGG GGCCGGUACAGAAGGUAGCAAUAUCGUGAGAUGGAGCAAAUCCUC-AAAGCUGGCC

AB001791_Ohmiya ACAUAGAUAAUCUGUCCUCAAC GUUGAGGGAGAGUCUAUGG GGCCAGUACAGAAGGUAGCAAUAUCGUGAGAUGGAGCAAAUCCUC-AAAGCUGGCC

AB001787_Itoh ACAUAGAUAAUCUGUCCUCAAC GUUGAGGGAGAGUCUAUGG GGCCAGUACAGAAGGUAGCAAUAUCGUGAGAUGGAGCAAAUCCUC-AAAGCUGGCC

AB285329_CPX0308 ACAUAGAUAAUCUGUCCUCAAC GUUAAGGAAGAGUCUAUGG GGCCAGUACAGAAGGUAGCAAUAUCGCGAGAUGGAGCAAAUCCUC-AAAGCUGGCC

D85708_GPIC ACAUAGAUAAUCUGUCCUCAAC GUUAAGGAAGAGUCUAUGG GGCCAGUACAGAAGGUAGCAAUAUCGCAAGAUGGAGCAAAUCCUC-AAAGCUGGCC

AE015925_GPIC ACAUAGAUAAUCUGUCCUCAAC GUUAAGGAAGAGUCUAUGG GGCCAGUACAGAAGGUAGCAAUAUCGCAAGAUGGAGCAAAUCCUC-AAAGCUGGCC

D85701_Fe_Baker ACAUAGAUAAUCUGCCCUCAAC GUUGGGGAAGAGUCUAUGG GGCCAGUACAGAAGGUAGCAAUAUCGUGAGAUGGAGCAAAUCCUC-AAAGCUGGCC

D85706_Fe_Cello ACAUAGAUAAUCUGCCCUCAAC GUUGGGGAAGAGUCUAUGG GGCCAGUACAGAAGGUAGCAAUAUCGUGAGAUGGAGCAAAUCCUC-AAAGCUGGCC

D85702_Fe/145 ACAUAGAUAAUCUGCCCUCAAC GUUGGGGAAGAGUCUAUGG GGCCAGUACAGAAGGUAGCAAUAUCGUGAGAUGGAGCAAAUCCUC-AAAGCUGGCC

AP006861_Fe/C-56 ACAUAGAUAAUCUGCCCUCAAC GUUGGGGAAGAGUCUAUGG GGCCAGUACAGAAGGUAGCAAUAUCGUGAGAUGGAGCAAAUCCUC-AAAGCUGGCC

D88317_E58 ACAUAGAUAAUUUG-UCUUAAC GUUAAGAGAAAGUCUGUGG GGGUAGUACAGAAGGUAGCAAGAUCGUGAGAUGGAGCAAAUCCUU-AAAGCUAUCC

U73785_Bo/E8 ACAUAGAUAAUUUGUUCUUAAC GUUAAGAGAAAGUCUGUGG GGGUAGUACAGAAGGUAGCAAGAUCGUGAGAUGGAGCAAAUCCUU-AAAGCUAUCC

U73782_BE ACAUAGAUAAUUUGUUCUUAAC GUUAAGAGAAAGUCUGUGG GGGUAGUACAGAAGGUAGCAAGAUCGUGAGAUGGAGCAAAUCCUU-AAAGCUAUCC

D85716_Ov/IPA ACAUAGAUAAUUUG-UCUUAAC GUUAAGAGAAAGUCUGUGG GGGUAGUACAGAAGGUAGCAAGAUCGUGAGAUGGAGCAAAUCCUU-AAAGCUAUCC

D85715_Maeda ACAUAGAUAAUUUG-UCUUAAC GUUAAGAGAAAGUCUGUGG GGGUAGUACAGAAGGUAGCAAGAUCGUGAGAUGGAGCAAAUCCUU-AAAGCUAUCC

D85714_Shizuoka ACAUAGAUAAUUUG-UCUUAAC GUUAAGAGAAAGUCUGUGG GGGUAGUACAGAAGGUAGCAAGAUCGUGAGAUGGAGCAAAUCCUU-AAAGCUAUCC

D85717_Koala_II ACAUAGAUAAUUGU-UCUUAAC GUUAAGAGAAAGUCUGUGG GGGUAGUACAGAAGGUAGCAAGAUCGUGAGAUGGAGCAAAUCCUU-AAAGCUAUCC

L06108_TW-183 ACAUAGAUAAUCUGCCCUCAAC GUUGAGGAAGAGUUUAUGC GGUUAGUACAGAAGGUAGCAAGAUCGUGAGAUGGAGCAAAUCCUA-AAAGCUAGCC

AE001363_CWL029 ACAUAGAUAAUCUGCCCUCAAC GUUGAGGAAGAGUUUAUGC GGUUAGUACAGAAGGUAGCAAGAUCGUGAGAUGGAGCAAAUCCUA-AAAGCUAGCC

DQ444323_WBB ACAUAGAUAAUCUGCCCUCAAC GUUGAGGAAGAGUUUAUGC GGUUAGUACAGAAGGUAGCAAGAUCGUGAGAUGGAGCAAAUCCUA-AAAGCUAACC

AF139200_CPXT1 ACAUAGAUAAUCUGCCCUCAAC GUUGAGGAAGAGUUUAUGC GGUUAGUACAGAAGGUAGCAAGAUCGUGAGAUGGAGCAAAUCCUA-AAAGCUAACC

U68426_N16 ACAUAGAUAAUCUGCCCUCAAC GUUGAGGAAGAGUUUAUGC GGUUAGUACAGAAGGUAGCAACAUCGUGAGAUGGAGCAAAUCCUA-AAAGCUAACC

FJ236984_LPCoLN ACAUAGAUAAUCUGCCCUCAAC GUUGAGGAAGAGUUUAUGC GGUUAGUACAGAAGGUAGCAAGAUCGUGAGAUGGAGCAAAUCCUA-AAAGCUAACC

D89067_A/HAR-13 GCAUAGAUAAUUUGUCCUUAAC GUUAAGGGAGAGUCUAUGU GGCCAGUACAGAAGGUGGCAAGAUCGCGAGAUGGAGCAAAUCCUC-AAAGCUGGCC

D85719__B/TW-5/OT GCAUAGAUAAUUUGUCCUUAAC GUUAAGGGAGAGUCUAUGU GGCCAGUACAGAAGGUGGCAAGAUCGCGAGAUGGAGCAAAUCCUC-AAAGCUGGCC

D85720_C/TW-3/OT GCAUAGAUAAUUUGUCCUUAAC GUUAAGGGAGAGUCUAUGU GGCCAGUACAGAAGGUGGCAAGAUCGCGAGAUGGAGCAAAUCCUC-AAAGCUGGCC

D85721_D/TW-3/CX GCAUAGAUAAUUUGUCCUUAAC GUUAAGGGAGAGUCUAUGU GGCCAGUACAGAAGGUGGCAAGAUCGCGAGAUGGAGCAAAUCCUC-AAAGCUGGCC

AE001273_D/UW-3/CX GCAUAGAUAAUUUGUCCUUAAC GUUAAGGGAGAGUCUAUGU GGCCAGUACAGAAGGUGGCAAGAUCGCGAGAUGGAGCAAAUCCUC-AAAGCUGGCC

D85722_E/UW-5/CX GCAUAGAUAAUUUGUCCUUAAC GUUAAGGGAGAGUCUAUGU GGCCAGUACAGAAGGUAGCAAGAUCGUGAGAUGGAGCAAAUCCUC-AAAGCUGGCC

DQ019301_F/IC/CAL3 GCAUAGAUAAUUUGUCCUUAAC GUUAAGGGAGAGUCUAUGU GGCCAGUACAGAAGGUAGCAAGAUCGUGAGAUGGAGCAAAUCCUC-AAAGCUGGCC

U68443_L2/434/BU GCAUAGAUAAUUUGUCCUUAAC GUUAAGGGAGAGUCUAUGU GGCCAGUACAGAAGGUAGCAAGAUCGUGAGAUGGAGCAAAUCCUC-AAAGCUGGCC

D85718_MoPn GCAUAGAUAAUUUGUCCUUAAC GUUAAGGGAGAGUCUAUGU GGCCAGUACAGAAGGUAGCAAGAUCGCGAGAUGGAGCAAAUCCUC-AAAGCUGGCC

U68437_SFPD GCAUAGAUAAUUUGUCCUUAAC GUUAAGGGAGAGUCUAUGU GGCCAGUACAGAAGGUAGCAAGAUCGCGAGAUGGAGCAAAUCCUC-AAAGCUGGCC

U73110_S45 GCAUAGAUAAUUUGUCUUUAAC GUUAAAGGGGAGUCUAUGU GGCCAGUACAGAAGGUAGCAAGAUCGUGAGAUGGAGCAAAUCCUU-AAAGCUGGCC

U68420_R22 GCAUAGAUAAUUUGUCUUUAAC GUUAAAGGGGAGUCUAUGU GGCCAGUACAGAAGGUAGCAAGAUCGUGAGAUGGAGCAAAUCCUU-AAAGCUGGCC

Y07556_Bn9 ACAUGGAUAACUUGCCUUUAAC GUUAAAGAGAGGUCCAUGG GGUCGGUACAGAAGGCAGCGAAGCCGUGAGGUGAAGCAAAUCCCAAAAAGCCGAUC

DQ309029_Seine ACAUGGAUAACUUGCCUUUAAC GUUAAAGAGAGGUCCAUGG GGUCGGUACAGAAGGCAGCGAAGCCGUGAGGUGAAGCAAAUCCCAAAAAGCCGAUC

AF042496_WSU_86-1044 ACAUGGGUAAUCUACCUUUAAC GUUAAAGAAGGGCCCACGG GGCAGGUACAGAAGGCAGCGAAACCGCGAGGUCAAGCAAAUCCUACGAAGCCUGGC

AF346001_2032/99 ACAUGGGUAAUCUACCUUUAAC GUUAAAGAGGGGCCCAUGG GGCAGGUACAGAAGGCAGCGAAACCGCGAGGUCAAGCAAAUCCUACAAAGCCUGUC

U68460_Z ACAUGAGUAACUUACCUCUUAC GUAGGAGAGAGGCUCAUGG GGUCGGUACAGAAGGCAGCGAAGCCGAAAGGUGAAGCAAAUCCCA-AAAGCUCGAU

AY140910_YaeL ACAUGAGUAACAUACCUCUUUC GAAAGAGAAUGACUCAUGA GGUCGGUACAGAAGGUAGCAAGACCGAAAGGUGGAGCAAAUCCCC-AAAGCCGAUC

J01695_E._coli GUCUGGGA-AACUGCCUGAUGG CCAUCGGAUGUGCCCAGAU GGCGCAUACAAAGAGAAGCGACCUCGCGAGAGCAAGCGGACCUCAUAAAGUGCGUC
