# Supplementary material for: Evolutionary Relationships among Chlamydophila abortus Variant Strains Inferred by rRNA Secondary Structure-Based Phylogeny
Source: PLoS One. 2011 May 24;6(5):e19813. doi: 10.1371/journal.pone.0019813 (PMC3101216; doi:10.1371/journal.pone.0019813)
Supplement: Figure S3 — 23S domain I rRNA secondary structure-based alignment of Chlamydophila abortus and other Chlamydiales sp. sequences (67 strains), created with the SINA Webaligner (SILVA LSU reference alignment [33]). The helices and multistem-loop in which the LLG/POS variant presents SNVs (H150, ML between H150 & H183, and H271, at positions 152, [181–182], and 273, respectively), as well as the hairpin-loop in which the FAG/VPG variant presents a SNV (HL of the H533 at position 547) are shown in A. The positions in which C. abortus and C. psittaci species present nucleotide differences (positions 18/H15, 132/H131, 147/H131, 152/H150, 157/H150, [181–182]/ML177–182, 240/H235, 297/H271, and 547/HL545–548) are shown in A and B. Helix numbering and nucleotide positions are according to the E. coli numbering system (Comparative RNA Web, CRW site [35]). Relevant positions are indicated in boldface and shaded with their paired base positions; the latter appear in normal font. Loops and bulges are indicated with grey letters. Alignments were used to generate the Tables 1 and 2 of the paper. (DOC) [file pone.0019813.s003.doc]

**Figure S3 A.**

**H150(150-158/168-176) ML between H271(271-297/341-366) H533(533-544/549-560)**

**H150 & H183 HL(545-548)**

**152 157 :** 169 174 **[181-182] 273 297:**341 364 **547**

U68445_B577 UUGUACGCUGAAUACAUAGGCGUAUAAGGCGAUAC- CGAUAACAUGGGA-UC-UUAAGU-UUUAGUC GACGAAA-AAAC-AAGA-GA-CUC-UAUUCG GUCGAGGACC-UAU--AACUUCUUCG-GAAGUC-AUGGUUGACGGC

EF486856_LLG UUAUACGCUGAAUACAUAGGCGUAUAAGGCGACAC- CGGUAACAUGGGA-UC-UUAAGU-UUUAGUC GACGAAA-AAAC-AAGA-GA-CUC-UAUUCG GUCGAAGACC-UAU--AACUUCUUCG-GAAGUC-AUGGUUGACGGC

EF486857_POS UUAUACGCUGAAUACAUAGGCGUAUAAGGCGACAC- CGGUAACAUGGGA-UC-UUAAGU-UUUAGUC GACGAAA-AAAC-AAGA-GA-CUC-UAUUCG GUCGAAGACC-UAU--AACUUCUUCG-GAAGUC-AUGGUUGACGGC

EF486853_FAS UUGUACGCUGAAUACAUAGGCGUAUAAGGCGAUAC- CGAUAACAUGGGA-UC-UUAAGU-UUUAGUC GACGAAA-AAAC-AAGA-GA-CUC-UAUUCG GUCGAAGACC-UAU--AACUUCUUCG-GAAGUC-AUGGUUGACGGC

EF486854_FAG UUGUACGCUGAAUACAUAGGCGUAUAAGGCGAUAC- CGAUAACAUGGGA-UC-UUAAGU-UUUAGUC GACGAAA-AAAC-AAGA-GA-CUC-UAUUCG GUCGAAGACC-UAU--AACUUCUUUG-GAAGUC-AUGGUUGACGGC

EF486855_VPG UUGUACGCUGAAUACAUAGGCGUAUAAGGCGAUAC- CGAUAACAUGGGA-UC-UUAAGU-UUUAGUC GACGAAA-AAAC-AAGA-GA-CUC-UAUUCG GUCGAAGACC-UAU--AACUUCUUUG-GAAGUC-AUGGUUGACGGC

CR848038_S263 UUGUACGCUGAAUACAUAGGCGUAUAAGGCGAUAC- CGAUAACAUGGGA-UC-UUAAGU-UUUAGUC GACGAAA-AAAC-AAGA-GA-CUC-UAUUCG GUCGAAGACC-UAU--AACUUCUUCG-GAAGUC-AUGGUUGACGGC

U76710|_EBA UUGUACGCUGAAUACAUAGGCGUAUAAGGCGAUAC- CGAUAACAUGGGA-UC-UUAAGU-UUUAGUC GACGAAA-AAAC-AAGA-GA-CUC-UAUUCG GUCGAAGACC-UAU--AACUUCUUCG-GAAGUC-AUGGUUGACGGC

U68444_A22 UUGUACGCUGAAUACAUAGGCGUAUAAGGCGAUAC- CGAUAACAUGGGA-UC-UUAAGU-UUUAGUC GACGAAA-AAAC-AAGA-GA-CUC-UAUUCG GUCGAAGACC-UAU--AACUUCUUCG-GAAGUC-AUGGUUGACGGC

U68446_OSP UUGUACGCUGAAUACAUAGGCGUAUAAGGCGAUAC- CGAUAACAUGGGA-UC-UUAAGU-UUUAGUC GACGAAA-AAAC-AAGA-GA-CUC-UAUUCG GUCGAAGACC-UAU--AACUUCUUCG-GAAGUC-AUGGUUGACGGC

U68447_6BC UUAUACGUUGAAUACAUAGGCGUAUAAGGCGACAC- CGAUAACAUGGGA-UC-UUAAGU-UUUAGUU GACGAAA-AAAC-AAGA-GA-CUC-UAUUCG GUCGAAGACC-UAU--AACUUCUUAG-GAAGUC-AUGGUUGACGGC

U68452_M56 UUAUACGUUGAAUACAUAGGCGUAUAAGGCGACAC- CGAUAACAUGGGA-UC-UUAAGU-UUUAGUU GACGAAA-AAAC-AAGA-GA-CUC-UAUUCG GUCGAAGACC-UAU--AACUUCUUAG-GAAGUC-AUGGUUGACGGC

AF481052_VS1 UUAUACGUUGAAUACAUAGGCGUAUAAGGCGACAC- CGAUAACAUGGGA-UC-UUAAGU-UUUAGUU GACGAAA-AAAC-AAGA-GA-CUC-UAUUCG GUCGAAGACC-UAU--AACUUCUUAG-GAAGUC-AUGGUUGACGGC

AF481049_VS225 UUAUACGUUGAAUACAUAGGCGUAUAAGGCGACAC- CGAUAACAUGGGA-UC-UUAAGU-UUUAGUU GACGAAA-AAAC-AAGA-GA-CUC-UAUUCG GUCGAAGACC-UAU--AACUUCUUAG-GAAGUC-AUGGUUGACGGC

U68454_MN UUAUACGUUGAAUACAUAGGCGUAUAAGGCGACAC- CGAUAACAUGGGA-UC-UUAAGU-UUUAGUU GACGAAA-AAAC-AAGA-GA-CUC-UAUUCG GUCGAAGACC-UAU--AACUUCUUAG-GAAGUC-AUGGUUGACGGC

U68453_MN_VR122 UUAUACGUUGAAUACAUAGGCGUAUAAGGCGACAC- CGAUAACAUGGGA-UC-UUAAGU-UUUAGUU GACGAAA-AAAC-AAGA-GA-CUC-UAUUCG GUCGAAGACC-UAU--AACUUCUUAG-GAAGUC-AUGGUUGACGGC

AF481051_MNRh UUAUACGUUGAAUACAUAGGCGUAUAAGGCGACAC- CGAUAACAUGGGA-UC-UUAAGU-UUUAGUU GACGAAA-AAAC-AAGA-GA-CUC-UAUUCG GUCGAAGACC-UAU--AACUUCUUAG-GAAGUC-AUGGUUGACGGC

AF481050_MNOs UUAUACGUUGAAUACAUAGGCGUAUAAGGCGACAC- CGAUAACAUGGGA-UC-UUAAGU-UUUAGUU GACGAAA-AAAC-AAGA-GA-CUC-UAUUCG GUCGAAGACC-UAU--AACUUCUUAG-GAAGUC-AUGGUUGACGGC

U68448_CP3 UUAUACGUUGAAUACAUAGGCGUAUAAGGCGACAC- CGAUAACAUGGGA-UC-UUAAGU-UUUAGUU GACGAAA-AAAC-AAGA-GA-CUC-UAUUCG GUCGAAGACC-UAU--AACUUCUUAG-GAAGUC-AUGGUUGACGGC

U68449_CT1 UUAUACGUUGAAUACAUAGACGUAUAAGGCGACAC- CGAUAACAUGGGA-UC-UUAAGU-UUUAGUU GACGAAA-AAAC-AAGA-GA-CUC-UAUUCG GUCGAAGACC-UAU--AACUUCUUAG-GAAGUC-AUGGUUGACGGC

U68450_GD UUAUACGUUGAAUACAUAGACGUAUAAGGCGACAC- CGAUAACAUGGGA-UC-UUAAGU-UUUAGUU GACGAAA-AAAC-AAGA-GA-CUC-UAUUCG GUCGAAGACC-UAU--AACUUCUUAG-GAAGUC-AUGGUUGACGGC

U68456_WC UUAUACGUUGAAUACAUAGACGUAUAAGGCGACAC- CGAUAACAUGGGA-UC-UUAAGU-UUUAGUU GACGAAA-AAAC-AAGA-GA-CUC-UAUUCG GUCGAAGACC-UAU--AACUUCUUAG-GAAGUC-AUGGUUGACGGC

U68455_Par1 UUAUACGUUGAAUACAUAGACGUAUAAGGCGACAC- CGAUAACAUGGGA-UC-UUAAGU-UUUAGUU GACGAAA-AAAC-AAGA-GA-CUC-UAUUCG GUCGAAGACC-UAU--AACUUCUUAG-GAAGUC-AUGGUUGACGGC

U68419_NJ1 UUAUACGUUGAAUACAUAGGCGUAUAAGGCGACAC- CGAUAACAUGGGA-UC-UUAAGU-UUUAGUU GACGAAA-AAAC-AAGA-GA-CUC-UAUUCG GUCGAAGACC-UAU--AACUUCUUAG-GAAGUC-AUGGUUGACGGC

AF481048_Daruma UUAUACGCUGAAUACAUAGGCGUAUAAGGCGACAC- CGAUAACAUGGGA-UC-UUAAGU-UUUAGUC GACGAAA-AAAC-AAGA-GA-CUC-UAUUCG GUCGAAGACC-UAU--AACUUCUUCG-GAAGUC-AUGGUUGACGGC

U68451_GPIC UUAUAUACUGAAUACAUAGGUAUAUAAAGCGACAC- CGAUAACAUGAGA-UC-UUAAGU-UUUAGUU AACGAAA-GAAC-AAGA-GA-CUC-UAUUCG GUCGGAGACC-UAU--AGCUUCCUCG-GAAGCC-AUGGUUGACGGC

AE015925_GPIC UUAUAUACUGAAUACAUAGGUAUAUAAAGCGACAC- CGAUAACAUGAGA-UC-UUAAGU-UUUAGUU AACGAAA-GAAC-AAGA-GA-CUC-UAUUCG GUCGGAGACC-UAU--AGCUUCCUCG-GAAGCC-AUGGUUGACGGC

U68457_FP_baker UUAUAUGCUGAAUACAUAGGCAUAUAAAGCGACAC- CGAUAACAUGGGA-UC-UUAAGU-UUUAGUU AACGAAA-AAAC-AAGA-GA-CUC-UAUUCG GUCGAAGACC-UAU-UAGCUUUUUCG-AAAGCA-AUGGUUGACGGC

U68458_FP_Cello UUAUAUGCUGAAUACAUAGGCAUAUAAAGCGACAC- CGAUAACAUGGGA-UC-UUAAGU-UUUAGUU AACGAAA-AAAC-AAGA-GA-CUC-UAUUCG GUCGAAGACC-UAU-UAGCUUUUUCG-AAAGCA-AUGGUUGACGGC

AP006861_Fe/C-56 UUAUAUGCUGAAUACAUAGGCAUAUAAAGCGACAC- CGAUAACAUGGGA-UC-UUAAGU-UUUAGUU AACGAAA-AAAC-AAGA-GA-CUC-UAUUCG GUCGAAGACC-UAU-UAGCUUUUUCG-AAAGCA-AUGGUUGACGGC

U68433_E58 UUAUACGCUGAAUCCAUAGGCGUAUAAGGCGAAAC- CGAUAACGUGAGA-UC-UUAAGU-UUUAGUU AACGAAA-AAAC-AAAA-GA-CUC-UAUUCG GUCGAAGACC-UAU--AACUC-UUCG--GAGUG-AUGGUUGACGGC

U68434_IPA UUAUACGCUGAAUCCAUAGGCGUAUAAGGCGAAAC- CGAUAACGUGAGA-UC-UUAAGU-UUUAGUU AACGAAA-AAAC-AAAA-GA-CUC-UAUUCG GUCGAAGACC-UAU--AACUC-UUCG--GAGUG-AUGGUUGACGGC

U68435_L71 UUAUACGCUGAAUCCAUAGGCGUAUAAGGCGAAAC- CGAUAACGUGAGA-UC-UUAAGU-UUUAGUU AACGAAA-AAAC-AAAA-GA-CUC-UAUUCG GUCGAAGACC-UAU--AACUC-UUCG--GAGUG-AUGGUUGACGGC

U68432_BP1 UUAUACGCUGAAUCCAUAGGCGUAUAAGGCGAAAC- CGAUAACGUGAGA-UC-UUAAGU-UUUAGUU AACGAAA-AAAC-AAAA-GA-CUC-UAUUCG GUCGAAGACC-UAU--AACUC-UUCG--GAGUG-AUGGUUGACGGC

U68431_1710S UUAUACGCUGAAUCCAUAGGCGUAUAAGGCGAAAC- CGAUAACGUGAGA-UC-UUAAGU-UUUAGUU AACGAAA-AAAC-AAAA-GA-CUC-UAUUCG GUCGAAGACC-UAU--AACUC-UUCG--GAGUG-AUGGUUGACGGC

U68439_Z UUAUACGCUGAAUCCAUAGGCGUAUAAGGCGAAAC- CGAUAACGUGAGA-UC-UUAAGU-UUUAGUU AACGAAA-AAAC-AAAA-GA-CUC-UAUUCG GUCGAAGACC-UAU--AACUC-UUCG--GAGUG-AUGGUUGACGGC

U76711_TW-183 UUAUAUGUUGAAUACAUAGGCAUAUAAGGCGACAC- CGAUAACAUGGGA-UC-UUAAGU-UUUAGUU AACGAAA-AAAC-AAAA-GA-CGC-UAAUCG GUCGGAGACC-UAU--AACUC-UUCG--GAGUA-AUGGUUGACGGC

AE017160_TW-183 UUAUAUGUUGAAUACAUAGGCAUAUAAGGCGACAC- CGAUAACAUGGGA-UC-UUAAGU-UUUAGUU AACGAAA-AAAC-AAAA-GA-CGC-UAAUCG GUCGGAGACC-UAU--AACUC-UUCG--GAGUA-AUGGUUGACGGC

U68422_CWL029 UUAUAUGUUGAAUACAUAGGCAUAUAAGGCGACAC- CGACAACAUGGGA-UC-UUAAGU-UUUAGUU AACGAAA-AAAC-AAAA-GA-CGC-UAAUCG GUCGGAGACC-UAU--AACUC-UUCG--GAGUA-AUGGUUGACGGC

U68423_CWL1011 UUAUAUGUUGAAUACAUAGGCAUAUAAGGCGACAC- CGAUAACAUGGGA-UC-UUAAGU-UUUAGUU AACGAAA-AAAC-AAAA-GA-CGC-UAAUCG GUCGGAGACC-UAU--AACUC-UUCG--GAGUA-AUGGUUGACGGC

U68424_FML12 UUAUAUGUUGAAUACAUAGGCAUAUAAGGCGACAC- CGAUAACAUGGGA-UC-UUAAGU-UUUAGUU AACGAAA-AAAC-AAAA-GA-CGC-UAAUCG GUCGGAGACC-UAU--AACUC-UUCG--GAGUA-AUGGUUGACGGC

U68425_FML16 UUAUAUGUUGAAUACAUAGGCAUAUAAGGCGACAC- CGAUAACAUGGGA-UC-UUAAGU-UUUAGUU AACGAAA-AAAC-AAAA-GA-CGC-UAAUCG GUCGGAGACC-UAU--AACUC-UUCG--GAGUA-AUGGUUGACGGC

U68421_CM1 UUAUAUGUUGAAUACAUAGGCAUAUAAGGCGACAC- CGAUAACAUGGGA-UC-UUAAGU-UUUAGUU AACGAAA-AAAC-AAAA-GA-CGC-UAAUCG GUCGGAGACC-UAU--AACUC-UUCG--GAGUA-AUGGUUGACGGC

U68426_N16 UUAUAUGCUGAAUACAUAGGCAUAUAAGGCGACAC- CGAUAACAUGGGA-UC-UUAAGU-UUUAGUU AACGAAA-AAAC-AAAA-GG-CAC-UAAUCG GUCGGAGACC-UAU--AACUC-UUCG--GAGUA-AUGGUUGACGGC

U68438_A/Har-13 UUGCAUGCUGAAUACAUAGGUAUGCAAAGCGACAC- UGAGGAUAAAGGA-UC-AGGACU-CCUAGUU GACGAAA-GGAGAGAAA-GA-CCGACC-UCA GUCGGAGACC-AAU--GGCCC-GUAA--GGGUC-AAGGUUGACGGC

CP000051_A/HAR-13 UUGCAUGCUGAAUACAUAGGUAUGCAAAGCGACAC- UGAGGAUAAAGGA-UC-AGGACU-CCUAGUU GACGAAA-GGAGAGAAA-GA-CCGACC-UCA GUCGGAGACC-AAU--GGCCC-GUAA--GGGUC-AAGGUUGACGGC

U68440_B/TW-5/OT UUGCAUGCUGAAUACAUAGGUAUGCAAAGCGACAC- UGAGGAUAAAGGA-UC-AGGACU-CCUAGUU GACGAAA-GGAGAGAAA-GA-CCGACC-UCA GUCGGAGACC-AAU--GGCCC-GUAA--GGGUC-AAGGUUGACGGC

U68441_D/UW-3/CX UUGCAUGCUGAAUACAUAGGUAUGCAAAGCGACAC- UGAGGAUAAAGGA-UC-AGGACU-CCUAGUU GACGAAA-GGAGAGAAA-GA-CCGACC-UCA GUCGGAGACC-AAU--GGCCC-GUAA--GGGUC-AAGGUUGACGGC

U68442_F/IC/CAL3 UUGCAUGCUGAAUACAUAGGUAUGCAAAGCGACAC- UGGGGAUAAAGGA-UC-AGAACU-CCUAGUU GACGAAA-GGAGAGAAA-GA-CCGACC-UCA GUCGGAGACC-AAU--GGCCC-GUAA--GGGUC-AAGGUUGACGGC

U68443_L2/434/BU UUGCAUGCUGAAUACAUAGGUAUGCAGAGCGACAC- UGAGGAUAAAGGA-UC-AGGACU-CCUAGUU GACGAAA-GGAGAGAAA-GA-CCGACC-UCA GUCGGAGACC-AAU--GGCCC-GUAA--GGGUC-AAGGUUGACGGC

U68436_MoPn UUGUAUACUGAAUACAUAGGUAUGCAAAGCAACAC- UGGGGAUAAAGGA-UC-AAGAUU-CCUAGUU GACGAAA-GGAGAUCAA-GA-CCGACC-UCA GUCGAAGACC-UAU--GUCCCUUUAACGGGGUC-GAGGUUGACGGC

U68437_SFPD UUGUAUACUGAAUACAUAGGUAUGCAAAGCAACAC- UGGGGAUAAAGGA-UC-AAGAUU-CCUAGUU GACGAAA-GGAGAUCAA-GA-CCGACC-UCA GUCGAAGACC-UAU--GUCCCUUUAACGGGGUC-GAGGUUGACGGC

AE002160_Nigg UUGUAUACUGAAUACAUAGGUAUGCAAAGCAACAC- UGGGGAUAAAGGA-UC-AAGAUU-CCUAGUU GACGAAA-GGAGAUCAA-GA-CCGACC-UCA GUCGAAGACC-UAU--GUCCCUUUAACGGGGUC-GAGGUUGACGGC

U73110_S45 UUGUAUGCUGAACACAUAGGCAUACAAAGCGACAC- UGAGGGAGAAGGA-UC-AGGACU-CCUAGUU GACGAAA-GGAGAGAUA-GA-CCGACC-UCG GUCGGAGACC-UAU--GGCCC-GCAA--GGGUU-AAGGUUGACGGC

U68420_R22 UUGUAUACUGAACACAUAGGUAUACAAAGCGACAC- UGAGGAAAAAGGA-UC-AGGACU-CCUAGUU GACGAAA-GGAGAGAAA-GA-CUGACC-UCA GUCGGAGACC-UAU--GGCCC-GUAA--GGGUU-AAGGUUGACGGC

DQ118376_MS04 UUGUAUACUGAACACAUAGGUAUGCAAAGCGACAC- UGAGGAAAAAGGA-UC-AGGACU-CCUAGUU GACGAAA-GGAGAGAAA-GA-CCGACC-UCA GUCGGAGACC-UAU--GGCCC-GUAA--GG----------------

AF481047_R19 UUGUAUACUGAACACAUAGGUAUACAAAGCGACAC- UGAGGAAAAAGGA-UC-AGGACU-CCUAGUU GACGAAA-GGAGAGAAA-GA-CUGACC-UCA GUCGGAGACC-UAU--GGCCC-GUAA--GGGUU-AAGGUUGACGGC

U68429_R27 UUGUAUACUGAACACAUAGGUAUACAAAGCGACAC- UGAGGAAAAAGGA-UC-AGGACU-CCUAGUU GACGAAA-GGAGAGAAA-GA-CCGACC-UCA GUCGGAGACC-UAU--GGCCC-GUAA--GGGUU-AAGGUUGACGGC

U68428_R24 UUGUAUACUGAACACAUAGGUAUACAAAGCGACAC- UGAGGAAAAAGGA-UC-AGGACU-CCUAGUU GACGAAA-GGAGAGAAA-GA-CCGACC-UCA GUCGGAGACC-UAU--GGCCC-GUAA--GGGUU-AAGGUUGACGGC

U68427_H5 UUGUAUACUGAACACAUAGGUAUACAAAGCGACAC- UGAGGAAAAAGGA-UC-AGGACU-CCUAGUU GACGAAA-GGAGAGAAA-GA-CCGACC-UCA GUCGGAGACC-UAC--GGCCC-GCAA--GGGUU-AAGGUUGACGGC

Y07555_Bn9 UCUUUAGCUGAAUACAUAGGCUAUAGAAGCGAUAC- CAGCCAAAAGC--UUU-GGAAAU-UCUAGUC GACGAAA-GAAU-GACC-AAA-CAAGG-CAG GUCGAAGGCC-UAU--GUCCUUUUU--AAGGAA-AUGGCUAACGGC

AF042496_WSU_86/1044 UCUUUACCUGAAUAAAUAGGGUAUAGAGGCGAGAC- CAGUCAAAGGA--UUG-GAGAUU-GUUAGUC GACGAAA-GCAA-GAUC-CGA-CAGGG-UUG GUCGGAGGCC-UAU--GAGCACUAA--GUGCUA-AUGGCUGACGGC

BX908798_UWE25 UCCUUAACUGAAUUCAUAGGUUAAGGAGGCGACAC- UCAUCACAAGG--CUA-GCGAAC-UUUAGCA UGCGAAA-AAGA-GAGC-UAG-UCAGA-UGA GUCGAAGGCC-UAU--GCCCAAUGAU-UUGGGA-AUGGCUGACGGC

AF346001_2032/99 UCUUUACCUGAAUAAAUAGGGUAUAGAGGCGAGAC- CAGUCAAAGGA--UUG-GAGAUU-GUUAGUC GACGAAA-GCAA-GAUC-CGA-CAGGA-UUG GUCGGAGGCC-UAU--GAGCACUAA--GUGCUA-AUGGCUGACGGC

AY184804_G817 UCUCUAUUUGAAUACAUAGGAUAGAGAAGCGAGAC- CUGUUAAAGGA--UUA-GGAAGU-AUUAGUC GACGAAA-AUAU-GACC-UAA-CAGGA-CAG GUCGGAGGCC-UAU--GAGCACUUA--GUGCUA-AUGGCUGACGGC

U68460_ Z UUCCUACCUGAAUACAUAGGGUUGGAAAGCAAUAC- UAGUCAUAAAG--CUU-AAGUGGACUUAGCA UGCAAAGAGAAA-GCUU-AAG-UGAGA-UUA GUCAGAGGCC-UAU--AUCCCUUUU--AGGGAA-AUGGCUGAUGGC

AY140910_YaeL UUUUUAACUGAAUUCAUAGGUUAAAAAAGCAA-AC- AAAUCAUAAAG--AAA-AUGUAAUCUUAACA UGUGAAAAGAAA-GCAU-GGA-CGAGA-UUU GUCAGAGGUC-UAU--ACUUUCUUUUAGAAAGC-AUGGCUGAUGGC

J01695_E.coli UCAUUAACUGAAUCCAUAGGUUAAUGAGGCGA-AC- GAGC-CUGAAU--CAGUGUGUGU-GUUAGUG CACAAAA-AUGC-ACAUGCUG-UGA-G-CUC GUGGGAGCAC---GC-------UUAG-------G-CGUGUGACUGC

**Figure S3 B.**

**H15(15-30/510-525) H131(131-137/142-148)**  **H235(235-246/252-262)**

**18 :** 522 **132 : 147 240**

U68445_B577 GAGUUAUUGGUGGAUG CUGAAACCAGUAGCUU GAUAG---AUUAAUCAU---CUAUC UCCCUGAGUAGCGGCGAGCGAAAGGGGA

EF486856_LLG GAGUUAUUGGUGGAUG CUGAAACCAGUAGCUU GAUAG---AUUAAUCAU---CUAUC UCCCUGAGUAGCGGCGAGCGAAAGGGGA

EF486857_POS GAGUUAUUGGUGGAUG CUGAAACCAGUAGCUU GAUAG---AUUAAUCAU---CUAUC UCCCUGAGUAGCGGCGAGCGAAAGGGGA

EF486853_FAS GAGUUAUUGGUGGAUG CUGAAACCAGUAGCUU GAUAG---AUUAAUCAU---CUAUC UCCCUGAGUAGCGGCGAGCGAAAGGGGA

EF486854_FAG GAGUUAUUGGUGGAUG CUGAAACCAGUAGCUU GAUAG---AUUAAUCAU---CUAUC UCCCUGAGUAGCGGCGAGCGAAAGGGGA

EF486855_VPG GAGUUAUUGGUGGAUG CUGAAACCAGUAGCUU GAUAG---AUUAAUCAU---CUAUC UCCCUGAGUAGCGGCGAGCGAAAGGGGA

CR848038_S263 GAGUUAUUGGUGGAUG CUGAAACCAGUAGCUU GAUAG---AUUAAUCAU---CUAUC UCCCUGAGUAGCGGCGAGCGAAAGGGGA

U76710|_EBA GAGUUAUUGGUGGAUG CUGAAACCAGUAGCUU GAUAG---AUUAAUCAU---CUAUC UCCCUGAGUAGCGGCGAGCGAAAGGGGA

U68444_A22 GAGUUAUUGGUGGAUG CUGAAACCAGUAGCUU GAUAG---AUUAAUCAU---CUAUC UCCCUGAGUAGCGGCGAGCGAAAGGGGA

U68446_OSP GAGUUAUUGGUGGAUG CUGAAACCAGUAGCUU GAUAG---AUUAAUCAU---CUAUC UCCCUGAGUAGCGGCGAGCGAAAGGGGA

U68447_6BC GAGCUAUUGGUGGAUG CUGAAACCAGUAGCUU GGUAG---AUUAAUCAU---CUACC UCCCUAAGUAGCGGCGAGCGAAAGGGGA

U68452_M56 GAGCUAUUGGUGGAUG CUGAAACCAGUAGCUU GGUAG---AUUAAUCAU---CUACC UCCCUAAGUAGCGGCGAGCGAAAGGGGA

AF481052_VS1 GAGCUAUUGGUGGAUG CUGAAACCAGUAGCUU GGUAG---AUUAAUCAU---CUACC UCCCUAAGUAGCGGCGAGCGAAAGGGGA

AF481049_VS225 GAGCUAUUGGUGGAUG CUGAAACCAGUAGCUU GGUAG---AUUAAUCAU---CUACC UCCCUAAGUAGCGGCGAGCGAAAGGGGA

U68454_MN GAGCUAUUGGUGGAUG CUGAAACCAGUAGCUU GGUAG---AUUAAUCAU---CUACC UCCCUAAGUAGCGGCGAGCGAAAAGGGA

U68453_MN_VR122 GAGCUAUUGGUGGAUG CUGAAACCAGUAGCUU GGUAG---AUUAAUCAU---CUACC UCCCUAAGUAGCGGCGAGCGAAAAGGGA

AF481051_MNRh GAGCUAUUGGUGGAUG CUGAAACCAGUAGCUU GGUAG---AUUAAUCAU---CUACC UCCCUAAGUAGCGGCGAGCGAAAAGGGA

AF481050_MNOs GAGCUAUUGGUGGAUG CUGAAACCAGUAGCUU GGUAG---AUUAAUCAU---CUACC UCCCUAAGUAGCGGCGAGCGAAAAGGGA

U68448_CP3 GAGCUAUUGGUGGAUG CUGAAACCAGUAGCUU GGUAG---AUUAAUCAU---CUACC UCCCUAAGUAGCGGCGAGCGAAAAGGGA

U68449_CT1 GAGCUAUUGGUGGAUG CUGAAACCAGUAGCUU GGUAG---AUUAAUCAU---CUACC UCCCUGAGUAGCGGCGAGCGAAAGGGGA

U68450_GD GAGCUAUUGGUGGAUG CUGAAACCAGUAGCUU GGUAG---AUUAAUCAU---CUACC UCCCUGAGUAGCGGCGAGCGAAAGGGGA

U68456_WC GAGCUAUUGGUGGAUG CUGAAACCAGUAGCUU GGUAG---AUUAAUCAU---CUACC UCCCUGAGUAGCGGCGAGCGAAAGGGGA

U68455_Par1 GAGCUAUUGGUGGAUG CUGAAACCAGUAGCUU GGUAG---AUUAAUCAU---CUACC UCCCUGAGUAGCGGCGAGCGAAAGGGGA

U68419_NJ1 GAGCUAUUGGUGGAUG CUGAAACCAGUAGCUU GGUAG---AUUAAUCAU---CUAUC UCCCUAAGUAGCGGCGAGCGAAAGGGGA

AF481048_Daruma GAGCUAUUGGUGGAUG CUGAAACCAGUAGCUU GAUAG---AUUAAUCAU---CUAUC UCCCUGAGUAGCGGCGAGCGAAAGGGGA

U68451_GPIC GAGCUAUUGGCGGAUG CUGAAACCAGUAGCUU GGUAG---AUUAAUCGU---CUACC UCCCUAAGUAGCGGCGAGCGAAAGGGGA

AE015925_GPIC GAGCUAUUGGCGGAUG CUGAAACCAGUAGCUU GGUAG---AUUAAUCGU---CUACC UCCCUAAGUAGCGGCGAGCGAAAGGGGA

U68457_FP_baker GAGCUAUUGGCGGAUG CUGAAACCAAUAGCUU GGUAG---AUUAAUCAU---CUACC UCCCUGAGUAGCGGCGAGCGAAAGGGGA

U68458_FP_Cello GAGCUAUUGGCGGAUG CUGAAACCAAUAGCUU GGUAG---AUUAAUCAU---CUACC UCCCUGAGUAGCGGCGAGCGAAAGGGGA

AP006861_Fe/C-56 GAGCUAUUGGCGGAUG CUGAAACCAAUAGCUU GGUAG---AUUAAUCAU---CUACC UCCCUGAGUAGCGGCGAGCGAAAGGGGA

U68433_E58 GAGCUAUUGGUGGAUG CUGAAACCAGUAGCUU GAUAG---AUUAAUAGU---CUAUC UCCCUGUGUAGCGGCGAGCGAAAGGGGA

U68434_IPA GAGCUAUUGGUGGAUG CUGAAACCAGUAGCUU GAUAG---AUUAAUAGU---CUAUC UCCCUGUGUAGCGGCGAGCGAAAGGGGA

U68435_L71 GAGCUAUUGGUGGAUG CUGAAACCAGUAGCUU GAUAG---AUUAAUAGU---CUAUC UCCCUGUGUAGCGGCGAGCGAAAGGGGA

U68432_BP1 GAGCUAUUGGUGGAUG CUGAAACCAGUAGCUU GAUAG---AUUAAUAGU---CUAUC UCCCUGUGUAGCGGCGAGCGAAAGGGGA

U68431_1710S GAGCUAUUGGUGGAUG CUGAAACCAGUAGCUU GAUAG---AUUAAUAGU---CUAUC UCCCUGUGUAGCGGCGAGCGAAAGGGGA

U68439_Z GAGCUAUUGGUGGAUG CUGAAACCAGUAGCUU GAUAG---AUUAAUAGU---CUAUC UCCCUGUGUAGCGGCGAGCGAAAGGGGA

U76711_TW-183 GAGCUAUUGGCGGAUG CUGAAACCAGUAGCUU GAUAG---ACUAAUAGU---CUAUC UCCCUGUGUAGCGGCGAGCGAAAGGGGA

AE017160_TW-183 GAGCUAUUGGCGGAUG CUGAAACCAGUAGCUU GAUAG---ACUAAUAGU---CUAUC UCCCUGUGUAGCGGCGAGCGAAAGGGGA

U68422_CWL029 GAGCUAUUGGCGGAUG CUGAAACCAGUAGCUU GAUAG---ACUAAUAGU---CUAUC UCCCUGUGUAGCGGCGAGCGAAAGGGGA

U68423_CWL1011 GAGCUAUUGGCGGAUG CUGAAACCAGUAGCUU GAUAG---ACUAAUAGU---CCAUC UCCCUGUGUAGCGGCGAGCGAAAGGGGA

U68424_FML12 GAGCUAUUGGCGGAUG CUGAAACCAGUAGCUU GAUAG---ACUAAUAGU---CUAUC UCCCUGUGUAGCGGCGAGCGAAAGGGGA

U68425_FML16 GAGCUAUUGGCGGAUG CUGAAACCAGUAGCUU GAUAG---ACUAAUAGU---CUAUC UCCCUGUGUAGCGGCGAGCGAAAGGGGA

U68421_CM1 GAGCUAUUGGCGGAUG CUGAAACCAGUAGCUU GAUAG---ACUAAUAGU---CUAUC UCCCUGUGUAGCGGCGAGCGAAAGGGGA

U68426_N16 GAGCUAUUGGCGGAUG CUGAAACCAGUAGCUU GAUAG---ACUAAUAGU---CUAUC UCCCUGUGUAGCGGCGAGCGAAAGGGGA

U68438_A/Har-13 GAGCUAUUGGUGGAUG CUGAAACCAGUAGCUU GGUAG---AGUAAUAGA---CUACC UCCCUGUGUAGCGGCGAGCGAAAGGGGA

CP000051_A/HAR-13 ---------------- CUGAAACCAGUAGCUU GGUAG---AGUAAUAGA---CUACC UCCCUGUGUAGCGGCGAGCGAAAGGGGA

U68440_B/TW-5/OT GAGCUAUUGGUGGAUG CUGAAACCAGUAGCUU GGUAG---AGUAAUAGA---CUACC UCCCUGUGUAGCGGCGAGCGAAAGGGGA

U68441_D/UW-3/CX GAGCUAUUGGUGGAUG CUGAAACCAGUAGCUU GGUAG---AGUAAUAGA---CUACC UCCCUGUGUAGCGGCGAGCGAAAGGGGA

U68442_F/IC/CAL3 GAGCUAUUGGUGGAUG CUGAAACCAGUAGCUU GGUAG---AGUAAUAGA---CUACC UCCCUGUGUAGCGGCGAGCGAAAGGGGA

U68443_L2/434/BU GAGCUAUUGGUGGAUG CUGAAACCAGUAGCUU GGUAG---AGUAAUAGA---CUACC UCCCUGUGUAGCGGCGAGCGAAAGGGGA

U68436_MoPn GAGCUAUUGGUGGAUG CUGAAACCAGUAGCUU GGUAG---AGUAAUAGU---CUACC UCCCUGUGUAGCGGCGAGCGAAAGGGGA

U68437_SFPD GAGCUAUUGGUGGAUG CUGAAACCAGUAGCUU GGUAG---AGUAAUAGU---CUACC UCCCUGUGUAGCGGCGAGCGAAAGGGGA

AE002160_Nigg GAGCUAUUGGUGGAUG CUGAAACCAGUAGCUU GGUAG---AGUAAUAGU---CUACC UCCCUGUGUAGCGGCGAGCGAAAGGGGA

U73110_S45 GAGCUAUUGGUGGAUG CUGAAACCAGUAGCUU GGUAG---AGUAAUAGA---CUACC UCCCUGUGUAGCGGCGAGCGAAAGGGGA

U68420_R22 GAGCUAUUGGUGGAUG CUGAAACCAAUAGCUU GGUAG---AGUAAUAGU---CUACC UCUCUGUGUAGCGGCGAGCGAAAGGGGA

DQ118376_MS04 GAGCUAUUGGUGGAUG CUGAAACCAAUAGCUU GGUAG---AGUAAUAGU---CUACC UCCCUGUGUAGCGGCGAGCGAAAGGGGA

AF481047_R19 GAGCUAUUGGUGGAUG CUGAAACCAAUAGCUU GGUAG---AGUAAUAGU---CUACC UCUCUGUGUAGCGGCGAGCGAAAGGGGA

U68429_R27 GAGCUAUUGGUGGAUG CUGAAACCAAUAGCUU GGUAG---AGUAAUAGU---CUACC UCCCUGUGUAGCGGCGAGCGAAAGGGGA

U68428_R24 GAGCUAUUGGUGGAUG CUGAAACCAAUAGCUU GGUAG---AGUAAUAGU---CUACC UCCCUGUGUAGCGGCGAGCGAAAGGGGA

U68427_H5 GAGCUAUUGGUGGAUG CUGAAACCAGUAGCUU GGUAG---AGUAAUAGU---CUACC UCCCUGUGUAGCGGCGAGCGAAAGGGGA

Y07555_Bn9 GGGCUGCUGGUGGAUG CUGAAACCAGCAGCUU AAUGGG--ACUAAU-CU--CCCAUU UCCCUAAGUAGCGGCGAGCGAAACGGGA

AF042496_WSU_86/1044 GAGCUGCUGGUGGAUG CUGAAACCAGCAGCUU AAUGGG--AUUAAC-AC--CCCAUU UCCCUGAGUAGCGGCGAGCGAAAUGGGA

BX908798_UWE25 GGGCUAUUGGUGGAUG CUGAAACCAAUAGCUU AACCAG--AGUAAU-GU--CUGGUU UCCCAUAGUAGCGGCGAGCGAAGUGGGA

AF346001_2032/99 GAGCUGCUGGUGGAUG CUGAAACCAGCAGCUU AAUGGG--AUUAAC-AC--CCCAUU UCCCUGAGUAGCGGCGAGCGAAAUGGGA

AY184804_G817 GAGCUACUGGUGGAUG CUGAAACCAGUAGCUU AAUGGG--AGUAGA-AU--CCCAUU UCCCGGAGUAGCGGCGAGCGAAAUGGGA

U68460_Z GAGCUAUUGGUGGAUG CUGAAACCAGUAGCUU AGUGGA--GUUAGU-CC--UCCACU UCCCGUAGUAGCGGCGAGCGAAGUGGGA

AY140910_YaeL ---------------- CUGAAACCAGCAGCUU AAUGGA--GCUAAU-UC--UCCAUU UCCCUUAGUAGCGGCGAGCGAAGCGGGA

J01695_E.coli GCGUACACGGUGGAUG CUGAAACCGUGUACGU AGUGUGU---UUCG----ACACACU UCCCCCAGUAGCGGCGAGCGAACGGGGA
