# Supplementary material for: Evolutionary Relationships among Chlamydophila abortus Variant Strains Inferred by rRNA Secondary Structure-Based Phylogeny
Source: PLoS One. 2011 May 24;6(5):e19813. doi: 10.1371/journal.pone.0019813 (PMC3101216; doi:10.1371/journal.pone.0019813)
Supplement: Figure S4 — A. 16S-23S rRNA intergenic spacer (IS) multiple sequence alignment of Chlamydophila abortus and other Chlamydiaceae sp. (57 strains), generated with CLUSTAL X (1.83) [32]. The IS sequences alignment of Parachlamydiaceae sp., Waddliaceae sp. and Simkaniaceae sp. strains, generated manually on the basis of the Chlamydiaceae sp. consensus sequence, is shown under the latter (see also [66]). The position in which the LLG/POS variant presents a SNV (position 79), and the positions in which C. abortus and C. psittaci species (shaded by yellow color) present interspecies differences (positions 49, 55–56, 185, 192–193, 198 and 204) are shaded. Relevant positions are indicated based on the 222 bp sequence of the C. abortus type strain B577T (U68445). Alignments were used to generate the Table 1 & 2 of the paper. B. Segment of stem region predicted to be formed between chlamydial 16S-23S rRNA IS and a complementary sequence of the 16S promoter [66]. The “-10 sequence” [68] is indicated with red letters. (DOC) [file pone.0019813.s004.doc]

**Figure S4 A.**

**49** **55-56** **79**  **185** **192-193** **198 204**

U68431_1710S -AAGGATAAGGAAAGCTATCAATTGTATAGCTTGACTAGGTTGGGCAAGCATTTT--------GCTGTGTATTCTATTTCTTTTGCTTTGTTAAGAG-------TGGTTTTCGTTACATTTAGTATTAATGATCAAGTATGTTAT-----GTAAATAATCATGGTAACAAGTATATTTTCACATATAATAATAGACGTTTAAGAATATCTGTCTTT--GGTGAAGTTATCTTGCATGGATCAAAAATT---- 225

U68435_L71 -AAGGATAAGGAAAGCTATCAATTGTATAGCTTGACTAGGTTGGGCAAGCATTTT--------GCTGTGTATTCTATTTCTTTTGCTTTGTTAAGAG-------TGGTTTTCGTTACATTTAGTATTAATGATCAAGTATGTTAT-----GTAAATAATCATGGTAACAAGTATATTTTCACATATAGTAATAGACGTTTAAGAATATCTGTCTTT--GGTGAAGTTATCTTGCATGGATCAAAAATT---- 225

U68432_BP1 -AAGGATAAGGAAAGCTATCAATTGTATAGCTTGACTAGGTTGGGCAAGCATTTT--------GCTGTGTATTCTATTTCTTTTGCTTTGTTAAGAG-------TGGTTTTCGTTACATTTAGTATTAATGATCAAGTATGTTAT-----GTAAATAATCATGGTAACAAGTATATTTTCACATATAATAATAGACGTTTAAGAATATCTGTCTTT--GGTGAAGTTATCTTGCATGGATCAAAAATT---- 225

U68433_E58 -AAGGATAAGGAAAGCTATCAATTGTATAGCTTGACTAGGTTGGGCAAGCATTTT--------GCTGTGTATTCTATTTCTTTTGCTTTGTTAAGAG-------TGGTTTTCGTTACATTTAGTATTAATGATCAAGTATGTTAT-----GTAAATAATCATGGTAACAAGTATATTTTCACATATAATAATAGACGTTTAAGAATATCTGTCTTT--GGTGAAGTTATCTTGCATGGATCAAAAATT---- 225

U68434_IPA -AAGGATAAGGAAAGCTATCAATTGTATAGCTTGACTAGGTTGGGCAAGCATTTT--------GCTGTGTATTCTATTTCTTTTGCTTTGTTAAGAG-------TGGTTTTCGTTACATTTAGTATTAATGATCAAGTATGTTAT-----GTAAATAATCATGGTAACAAGTATATTTTCACATATAATAATAGACGTTTAAGAATATCTGTCTTT--GGTGAAGTTATCTTGCATGGATCAAAAATT---- 225

U68439_Z -AAGGATAAGGAAAGCTATCAATTGTATAGCTTGACTAGGTTGGGCAAGCATTTT--------GCTGTGTATTCTATTTCTTTTGCTTTGTTAAGAG-------TGGTTTTCGTTACATTTAGTATTAATGATCAAGTATGTTAT-----GTAAATAATCATGGTAACAAGTATATTTTCACATATAATAATAGACGTTTAAGAATATCTGTCTTT--GGTGAAGTTATCTTGCATGGATCAAAAATT---- 225

U76711_TW-183 TAAGGACAAGGAAGGTTGTT--TTTAACAACCCGACTAGGTTGGGCAAGTATTTTAT------ATTCCGCATTCTATTTCTTTTGCATTGTTAAG-G-------TTGTTTTCAAAACATTCAGTAT--ATGATCAAGTATGTTAT-----GTAAATAATCATGGTAACAAGTAT-TTTTCACATATAATAATAGACGTTTAAGAATATCTGTCTTT-AGGTGAAGTTAACTTGCATGGATCAAAAATT---- 223

U68422_CWL029 TAAGGACAAGGAAGGTTGTT--TTTAACAACCCGACTAGGTTGGGCAAGTATTTTAT------ATTCCGCATTCTATTTCTTTTGCATTGTTAAG-G-------TTGTTTTCAAAACATTCAGTAT--ATGATCAAGTATGTTAT-----GTAAATAATCATGGTAACAAGTAT-TTTTCACATATAATAATAGACGTTTAAGAATATCTGTCTTT-AGGTGAAGTTAACTTGCATGGATCAAAAATT---- 223

U68423_CWL1011 TAAGGACAAGGAAGGTTGTT--TTTAACAACCCGACTAGGTTGGGCAAGTATTTTAT------ATTCCGCATTCTATTTCTTTTGCATTGTTAAG-G-------TTGTTTTCAAAACATTCAGTAT--ATGATCAAGTATGTTAT-----GTAAATAATCATGGTAACAAGTAT-TTTTCACATATAATAATAGACGTTTAAGAATATCTGTCTTT-AGGTGAAGTTAACTTGCATGGATCAAAAATT---- 223

U68424_FML12 TAAGGACAAGGAAGGTTGTT--TTTAACAACCCGACTAGGTTGGGCAAGTATTTTAT------ATTCCGCATTCTATTTCTTTTGCATTGTTAAG-G-------TTGTTTTCAAAACATTCAGTAT--ATGATCAAGTATGTTAT-----GTAAATAATCATGGTAACAAGTAT-TTTTCACATATAATAATAGACGTTTAAGAATATCTGTCTTT-AGGTGAAGTTAACTTGCATGGATCAAAAATT---- 223

U68425_FML16 TAAGGACAAGGAAGGTTGTT--TTTAACAACCCGACTAGGTTGGGCAAGTATTTTAT------ATTCCGCATTCTATTTCTTTTGCATTGTTAAG-G-------TTGTTTTCAAAACATTCAGTAT--ATGATCAAGTATGTTAT-----GTAAATAATCATGGTAACAAGTAT-TTTTCACATATAATAATAGACGTTTAAGAATATCTGTCTTT-AGGTGAAGTTAACTTGCATGGATCAAAAATT---- 223

U68421_CM1 -AAGGACAAGGAAGGTTGTT--TTTAACAACCCGACTAGGTTGGGCAAGTATTTTAT------ATTCCGCATTCTATTTCTTTTGCATTGTTAAG-G-------TTGTTTTCAAAACATTCAGTAT--ATGATCAAGTATGTTAT-----GTAAATAATCATGGTAACAAGTAT-TTTTCACATATAATAATAGACGTTTAAGAATATCTGTCTTT-AGGTGAAGTTAACTTGCATGGATCAAAAATT---- 222

U68426_N16 TAAGGACAAGGAAGGTTGTT--TTTAACAACCTGACTAGGTTGGGCAAGTATTTTAT------GTTCCGCATTCTATTTCTTTTGCATTGTTAAG-G-------TTGTTTTCAAAACATTCAGTAT--ATGATCAAGTATGTTAT-----GTAAATAATCATGGTAACAAGTAT-TTTTCACATATAATAATAGACGTTTAAGAATATCTGTCTTT-AGGTGAAGTTAACTTGCATGGATCAAAAATT---- 223

U68451_GPIC -AAGGATAAGGATGGCCGTCT-TAGGATGGTTTGACTAGGTTGGGCAAGCGTCTTTGA----AAACTTGTATTCTATTTCTTTTGCATTGTTAAGCGC------TCGTTTCCAAAACATTCAGTTT--ACGATCAAGTATGTTAT-----GTAAATAAT-ATGGTAACAAGTAA--ATTCACATATAATAATAGACGTTTAAGAATATCTGTCTTT--GGTGAAGTTAACTTGCATGGATCAATAATT---- 224

AE015925_GPIC TAAGGATAAGGATGGCCGTCT-TAGGATGGTTTGACTAGGTTGGGCAAGCGTCTTTGA----AAACTTGTATTCTATTTCTTTTGCATTGTTAAGCGC------TCGTTTCCAAAACATTCAGTTT--ACGATCAAGTATGTTAT-----GTAAATAAT-ATGGTAACAAGTAA--ATTCACATATAATAATAGACGTTTAAGAATATCTGTCTTT--GGTGAAGTTAACTTGCATGGATCAATAAT----- 224

U68457_FP baker -AAGGATAAGGATAACCGTTT-TAGGACGGTTTGACTAGGTTGGGCAAGCATTTTTGA----AAACTTGTATTCTATTTCTTTTGCGTTGTTAAGCG-------TGGGTTACAAAACATTCAGTTT--ACGATCAAGTATGTTAT-----GTAAATAAT-ATGGTAACAAGTAA--ATTCACATATAATAATAGACGTTTAAGAATATATGTCTTT-AGGTGAAGTTAACTTGCATGGATCAATAAT----- 223

AP006861_Fe/C-56 TAAGGATAAGGATAACCGTTT-TAGGACGGTTTGACTAGGTTGGGCAAGCATTTTTGA----AAACTTGTATTCTATTTCTTTTGCGTTGTTAAGCG-------TGGGTTACAAAACATTCAGTTT--ACGATCAAGTATGTTAT-----GTAAATAAT-ATGGTAACAAGTAA--ATTCACATATAATAATAGACGTTTAAGAATATATGTCTTT-AGGTGAAGTTAACTTGCATGGATCAATAAT----- 224

U68458_FP Cello ---GGATAAGGATAACCGTTT-TAGGACGGTTTGACTAGGTTGGGCAAGCATTTTTGA----AAACTTGTATTCTATTTCTTTTGCGTTGTTAAGCG-------TGGGTTACAAAACATTCAGTTT--ACGATCAAGTATGTTAT-----GTAAATAAT-ATGGTAACAAGTAA--ATTCACATATAATAATAGACGTTTAAGAATATATGTCTTT-AGGTGAAGTTAACTTGCATGGATCAATAATT---- 222

U68447_6BC -AAGGATAAGGATAACTGTCT-TAGGACGGTTTGACTAGGTTGGGCAAGCGTTTTTTT----AATCTTGTATTCTATTTCTTTTGCATTGTTAAGCG-------TTGTTTCCAAAACATTTAGTTT--ACGATCAAGTATGTTAT-----GTAAATAAT-ATGGTAACAAGTAA--ATTCACATATAATAATAGACGTTTAAGAATATATGTCTTT-AGGTGATGTTAACTTGCATGGATCAATAATT---- 224

U68452_M56 -AAGGATAAGGATAACTGTCT-TAGGACGGTTTGACTAGGTTGGGCAAGCGTTTTTTT----AATCTTGTATTCTATTTCTTTTGCATTGTTAAGCG-------TTGTTTCCAAAACATTTAGTTT--ACGATCAAGTATGTTAT-----GTAAATAAT-ATGGTAACAAGTAA--ATTCACATATAATAATAGACGTTTAAGAATATATGTCTTT-AGGTGATGTTAACTTGCATGGATCAATAATT---- 224

AF481052_VS1 -AAGGATAAGGATAACTGTCT-TAGGACGGTTTGACTAGGTTGGGCAAGCGTTTTTTT----AATCTTGTATTCTATTTCTTTTGCATTGTTAAGCG-------TTGTTTCCAAAACATTTAGTTT--ACGATCAAGTATGTTAT-----GTAAATAAT-ATGGTAACAAGTAA--ATTCACATATAATAATAGACGTTTAAGAATATATGTCTTT-AGGTGATGTTAACTTGCATGGATCAATAATT---- 224

AF481049_VS225 -AAGGATAAGGATAACTGTCT-TAGGACGGTTTGACTAGGTTGGGCAAGCGTTTTTTT----AATCTTGTATTCTATTTCTTTTGCATTGTTAAGCG-------TTGTTTCCAAAACATTTAGTTT--ACGATCAAGTATGTTAT-----GTAAATAAT-ATGGTAACAAGTAA--ATTCACATATAATAATAGACGTTTAAGAATATATGTCTTT-AGGTGATGTTAACTTGCATGGATCAATAATT---- 224

U68454_MN -AAGGATAAGGATAACTGTCT-TAGGACGGTTTGACTAGGTTGGGCAAGCGTTTTTT-----AATCTTGTATTCTATTTCTTTTGCATTGTTAAGTG-------TTGTTTCCAAAACATTTAGTTT--ACGATCAAGTATGTTAT-----GTAAATAAT-ATGGTAACAAGTAA--ATTCACATATAATAATAGACGTTTAAGAATATATGTCTTT-AGGTGATGTTAACTTGCATGGATCAATAATT---- 223

U68448_CP3 -AAGGATAAGGATAACTGTCT-TAGGACGGTTTGACTAGGTTGGGCAAGCGTTTTTT-----AATCTTGTATTCTATTTCTTTTGCATTGTTAAGTG-------TTGTTTCCAAAACATTTAGTTT--ACGATCAATTATGTTAT-----GTAAATAAT-ATGGTAACAAGTAA--ATTCACATATAATAATAGACGTTTAAGAATATATGTCTTT-AGGTGATGTTAACTTGCATGGATCAATAATT---- 223

U68453_MN VR122 -AAGGATAAGGATAACTGTCT-TAGGACGGTTTGACTAGGTTGGGCAAGCGTTTTTT-----AATCTTGTATTCTATTTCTTTTGCATTGTTAAGTG-------TTGTTTCCAAAACATTTAGTTT--ACGATCAAGTATGTTAT-----GTAAATAAT-ATGGTAACAAGTAA--ATTCACATATAATAATAGACGTTTAAGAATATATGTCTTT-AGGTGATGTTAACTTGCATGGATCAATAATT---- 223

AF481051_MNRh -AAGGATAAGGATAACTGTCT-TAGGACGGTTTGACTAGGTTGGGCAAGCGTTTTTT-----AATCTTGTATTCTATTTCTTTTGCATTGTTAAGTG-------TTGTTTCCAAAACATTTAGTTT--ACGATCAAGTATGTTAT-----GTAAATAAT-ATGGTAACAAGTAA--ATTCACATATAATAATAGACGTTTAAGAATATATGTCTTT-AGGTGATGTTAACTTGCATGGATCAATAATT---- 223

AF481050_MNOs -AAGGATAAGGATAACTGTCT-TAGGACGGTTTGACTAGGTTGGGCAAGCGTTTTTT-----AATCTTGTATTCTATTTCTTTTGCATTGTTAAGTG-------TTGTTTCCAAAACATTTAGTTT--ACGATCAAGTATGTTAT-----GTAAATAAT-ATGGTAACAAGTAA--ATTCACATATAATAATAGACGTTTAAGAATATATGTCTTT-AGGTGATGTTAACTTGCATGGATCAATAATT---- 223

U68449_CT1 -AAGGATAAGGATAACTGTCT-TAGGACGGTTTGACTAGGTTGGGCAAGCGTTTTTTT----AATCTTGTATTCTATTTCTTTTGCATTGTTAAGCG-------TTGTTTCCAAAACATTTAGTTT--ACGATCAAGTATGTTAT-----GTAAATAAT-ATGGTAACAAGTAA--ATTCACATATAATAATAGACGTTTAAGAATATATGTCTTT-AGGTGAAGTTAACTTGCATGGATCAATAATT---- 224

U68419_NJ1 -AAGGATAAGGATAACTGTCT-TAGGACGGTTTGACTAGGTTGGGCAAGCGTTTTTTT----AATCTTGTATTCTATTTCTTTTGCATTGTTAAGCG-------TTGTTTCCAAAACATTTAGTTT--ACGATCAAGTATGTTAT-----GTAAATAAT-ATGGTAACAAGTAA--ATTCACATATAATAATAGACGTTTAAGAATATATGTCTTT-AGGTGAAGTTAACTTGCGTGGATCAATAATT---- 224

U68450_GD -AAGGATAAGGATAACTGTCT-TAGGACGGTTTGACTAGGTTGGGCAAGCGTTTTTTT----AATCTTGTATTCTATTTCTTTTGCATTGTTAAGCG-------TTGTTTCCAAAACATTTAGTTT--ACGATCAAGTATGTTAT-----GTAAATAAT-ATGGTAACAAGTAA--ATTCACATATAATAATAGACGTTTAAGAATATATGTCTTT-AGGTGAAGTTAACTTGCATGGATCAATAATT---- 224

U68456_WC -AAGGATAAGGATAACTGTCT-TAGGACGGTTTGACTAGGTTGGGCAAGCGTTTTTTT----AATCTTGTATTCTATTTCTTTTGCATTGTTAAGCG-------TTGTTTCCAAAACATTTAGTTT--ACGATCAAGTATGTTAT-----GTAAATAAT-ATGGTAACAAGTAA--ATTCACATATAATAATAGACGTTTAAGAATATATGTCTTT-AGGTGAAGTTAACTTGCATGGATCAATAATT---- 224

U68455_Par1 -AAGGATAAGGATAACTGTCT-TAGGACGGTTTGACTAGGTTGGGCAAGCGTTTTTTT----AATCTTGTATTCTATTTCTTTTGCATTGTTAAGCG-------TTGTTTCCAAAACATTTAGTTT--ACGATCAAGTATGTTAT-----GTAAATAAT-ATGGTAACAAGTAA--ATTCACATATAATAATAGACGTTTAAGAATATATGTCTTT-AGGTGAAGTTAACTTGCATGGATCAATAATT---- 224

EF486856_LLG TAAGGATAAGGATAACTGTCT-TAGGACGGTTTGACTAGGTTGGGCAAGCATTTTTT-----AATCTTGTATTCTATTTCTTTTGTATTGTTAAGCG-------TTGTTTCCAAAACATTTAGTTT--ACGATCAAGTATGTTAT-----GTAAATAAT-ATGGTAACAAGTAA--ATTCACATATAATAATAGACGTTTAAGAATATCTGTCTTT--GGTGAAGTTAATTTGCATGGATCAATAATT---- 223

EF486857_POS TAAGGATAAGGATAACTGTCT-TAGGACGGTTTGACTAGGTTGGGCAAGCATTTTTT-----AATCTTGTATTCTATTTCTTTTGTATTGTTAAGCG-------TTGTTTCCAAAACATTTAGTTT--ACGATCAAGTATGTTAT-----GTAAATAAT-ATGGTAACAAGTAA--ATTCACATATAATAATAGACGTTTAAGAATATCTGTCTTT--GGTGAAGTTAATTTGCATGGATCAATAATT---- 223

EF486853_FAS TAAGGATAAGGATAACTGTCT-TAGGACGGTTTGACTAGGTTGGGCAAGCATTTTTT-----AATCTTGTATTCTATTTCTTTTGCATTGTTAAGCG-------TTGTTTCCAAAACATTTAGTTT--ACGATCAAGTATGTTAT-----GTAAATAAT-ATGGTAACAAGTAA--ATTCACATATAATAATAGACGTTTAAGAATATCTGTCTTT--GGTGAAGTTAATTTGCATGGATCAATAATT---- 223

CR848038_S26/3 TAAGGATAAGGATAACTGTCT-TAGGACGGTTTGACTAGGTTGGGCAAGCATTTTTT-----AATCTTGTATTCTATTTCTTTTGCATTGTTAAGCG-------TTGTTTCCAAAACATTTAGTTT--ACGATCAAGTATGTTAT-----GTAAATAAT-ATGGTAACAAGTAA--ATTCACATATAATAATAGACGTTTAAGAATATCTGTCTTT--GGTGAAGTTAATTTGCATGGATCAATAAT----- 222

EF486854_FAG TAAGGATAAGGATAACTGTCT-TAGGACGGTTTGACTAGGTTGGGCAAGCATTTTTT-----AATCTTGTATTCTATTTCTTTTGCATTGTTAAGCG-------TTGTTTCCAAAACATTTAGTTT--ACGATCAAGTATGTTAT-----GTAAATAAT-ATGGTAACAAGTAA--ATTCACATATAATAATAGACGTTTAAGAATATCTGTCTTT--GGTGAAGTTAATTTGCATGGATCAATAATT---- 223

EF486855_VPG TAAGGATAAGGATAACTGTCT-TAGGACGGTTTGACTAGGTTGGGCAAGCATTTTTT-----AATCTTGTATTCTATTTCTTTTGCATTGTTAAGCG-------TTGTTTCCAAAACATTTAGTTT--ACGATCAAGTATGTTAT-----GTAAATAAT-ATGGTAACAAGTAA--ATTCACATATAATAATAGACGTTTAAGAATATCTGTCTTT--GGTGAAGTTAATTTGCATGGATCAATAATT---- 223

U76710_EBA -AAGGATAAGGATAACTGTCT-TAGGACGGTTTGACTAGGTTGGGCAAGCATTTTTT-----AATCTTGTATTCTATTTCTTTTGCATTGTTAAGCG-------TTGTTTCCAAAACATTTAGTTT--ACGATCAAGTATGTTAT-----GTAAATAAT-ATGGTAACAAGTAA--ATTCACATATAATAATAGACGTTTAAGAATATCTGTCTTT--GGTGAAGTTAATTTGCATGGATCAATAATT---- 222

U68446_OSP -AAGGATAAGGATAACTGTCT-TAGGACGGTTTGACTAGGTTGGGCAAGCATTTTTT-----AATCTTGTATTCTATTTCTTTTGCATTGTTAAGCG-------TTGTTTCCAAAACATTTAGTTT--ACGATCAAGTATGTTAT-----GTAAATAAT-ATGGTAACAAGTAA--ATTCACATATAATAATAGACGTTTAAGAATATCTGTCTTT--GGTGAAGTTAATTTGCATGGATCAATAATT---- 222

U68444_A22 -AAGGATAAGGATAACTGTCT-TAGGACGGTTTGACTAGGTTGGGCAAGCATTTTTT-----AATCTTGTATTCTATTTCTTTTGCATTGTTAAGCG-------TTGTTTCCAAAACATTTAGTTT--ACGATCAAGTATGTTAT-----GTAAATAAT-ATGGTAACAAGTAA--ATTCACATATAATAATAGACGTTTAAGAATATCTGTCTTT--GGTGAAGTTAATTTGCATGGATCAATAATT---- 222

U68445_B577 -AAGGATAAGGATAACTGTCT-TAGGACGGTTTGACTAGGTTGGGCAAGCATTTTTT-----AATCTTGTATTCTATTTCTTTTGCATTGTTAAGCG-------TTGTTTCCAAAACATTTAGTTT--ACGATCAAGTATGTTAT-----GTAAATAAT-ATGGTAACAAGTAA--ATTCACATATAATAATAGACGTTTAAGAATATCTGTCTTT--GGTGAAGTTAATTTGCATGGATCAATAATT---- 222

EF165622_1V -AAGGATAAGGATAACTGTCT-TAGGACGGTTTGACTAGGTTGGGCAAGCATTTTTT-----AATCTTGTATTCTATTTCTTTTGCATTGTTAAGCG-------TTGTTTCCAAAACATTTAGTTT--ACGATCAAGTATGTTAT-----GTAAATAAT-ATGGTAACAAGTAA--ATTCACATATAATAATAGACGTTTAAGAATATCTGTCTTT--GGTGAAGTTAACTTGCATGGATCAATAATT---- 222

AJ310736_84/2334 -AAGGATAAGGATAACTGTCT-TAGGACGGTTTGACTAGGTTGGGCAAGCATTTTTT-----AATCTTGTATTCTATTTCTTTTGCATTGTTAAGCG-------TTGTTTCCAAAACATTTAGTTT--ACGATCAAGTATGTTAT-----GTAAATAAT-ATGGTAACAAGTAA--ATTCACATATAATAATAGACGTTTAAGAATATCTGTCTTT--GGTGAAGTTAACTTGCATGGATCAATAATT---- 222

AF481048_Daruma -AAGGATAAGGATAACTGTCT-TAGGACGGTTTGACTAGGTTGGGCAAGCATTTTTT-----AATCTTGTATTCTATTTCTTTTGCATTGTTAAGCG-------TTGTTTCCAAAACATTTAGTTT--ACGATCAAGTATGTTAT-----GTAAATAAT-ATGGTAACAAGTAA--ATTCACATATAATAATAGACGTTTAAGAATATCTGTCTTT--GGTGAAGTTAACTTGCATGGATCAATAATT---- 222

U68438_A/Har-13 -AAGGATAAGGAAGAA-GCCTGAGAGGGTTTCTGACTAGGTTGGGCAAGCGTTTATATGTAAGAGCAAGCATTCTATTTCATTTGTGTTGTTAAGAGTAGCGCGGTGAGGACGAGACATATAGTTT--GTGATCAAGTATGTTATTGTAAAGAAATAATCATGGTAACAAGTAT-ATTTCACGCATAATAATAGACGTTTAAGAGTATTTGTCTTTTAGGTGAAGT--GCTTGCATGGATCTATAGAAAT-- 243

U68440_B/TW-5/OT -AAGGATAAGGAAGAA-GCCTGAGAGGGTTTCTGACTAGGTTGGGCAAGCGTTTATATGTAAGAGCAAGCATTCTATTTCATTTGTGTTGTTAAGAGTAGCGCGGTGAGGACGAGACATATAGTTT--GTGATCAAGTATGTTATTGTAAAGAAATAATCATGGTAACAAGTAT-ATTTCACGCATAATAATAGACGTTTAAGAGTATTTGTCTTTTAGGTGAAGT--GCTTGCATGGATCTATAGAAAT-- 243

U68441_D/UW-3/CX -AAGGATAAGGAAGAA-GCCTGAGAAGGTTTCTGACTAGGTTGGGCAAGCGTTTATATGTAAGAGCAAGCATTCTATTTCATTTGTGTTGTTAAGAGTAGCGCGGTGAGGACGAGACATATAGTTT--GTGATCAAGTATGTTATTGTAAAGAAATAATCATGGTAACAAGTAT-ATTTCACGCATAATAATAGACGTTTAAGAGTATTTGTCTTTTAGGTGAAGT--GCTTGCATGGATCTATAGAAAT-- 243

U68442_F/IC/CAL3 -AAGGATAAGGAAGAA-GCCTGAGAAGGTTTCTGACTAGGTTGGGCAAGCATTTATATGTAAGAGCAAGCATTCTATTTCATTTGTGTTGTTAAGAGTAGCGCGGTGAGGACGAGACATATAGTTT--GTGATCAAGTATGTTATTGTAAAGAAATAATCATGGTAACAAGTAT-ATTTCACGCATAATAATAGACGTTTAAGAGTATTTGTCTTTTAGGTGAAGT--GCTTGCATGGATCTATAGAAAT-- 243

U68443_L2/434/BU -AAGGATAAGGAAGAA-GCCTGAGAAGGTTTCTGACTAGGTTGGGCAAGCATTTATATGTAAGAGCAAGCATTCTATTTCATTTGTGTTGTTAAGAGTAGCGTGGTGAGGACGAGACATATAGTTT--GTGATCAAGTATGTTATTGTAAAGAAATAATCATGGTAACAAGTAT-ATTTCACGCATAATAATAGACGTTTAAGAGTATTTGTCTTTTAGGTGAAGT--GCTTGCATGGATCTATAGAAAT-- 243

U73110_S45 -AAGGATAAGGAAGAA-GCCTGAGAAGGTTTCTGACTAGGTTGGGCAAGCGTTTATACGTAAGAGCGGGCATTCTATTTCATTTGCATTGTTAAG-GTAGCG-GAAGAGGACGAGACATATAGTTT--GTGATCAAGTATGTTAT-GTAAAGAAAAAATCATGGTAACAAGTAT-AATTCACGCATAATAATAGACGTTTAAGAGTATTTGTCTTT-AGGTGAAGT--ACTTGCATGGATCTATGAGAAAAA 241

U68420_R22 -AAGGATAAGGAAGAA-GCCTGAAAAGGTTTCTGACTAGGTTGGGCAAGCATTTATACGTAAGGGCGAGCATTCTATTTCATTTGCATTGTTAAG-GTAGCT-GAAGAGGACGAGACATATAGTTT--ATGATCAAGTATGTTAT-GTAAAGAAA--ATCATGGTAACAAGTAT-ATTTCACGCATAATAATAGACGTTTAAGAGTATTTGTCTTT-AGGTGAAGT--ACTTGCATGGATCTATGAGAAAT- 238

U68436_MoPn -AAGGATAAGGAAGAA-GCCTGAGAAGGTTTCTTACTAGGTTGAGCAAGCATTTATATGTAAGAGCAGGCATTCTATTTCATTTGCGTTGTTAAG-GTGGCGCGAAGAGGACGAAACATACAGTTT--GTGATCAAGTATGTTATTGTAAAGAAATAATCATGGTAACAAGTAT-AATTCACGCATAATAATAGACGTTTAAGAGTATTTGTCTTT-AGGTGAAGT--ACTTGCATGGATCTATGAAATT-- 241

U68437_SFPD -AAGGATAAGGAAGAA-GCCTGAGAAGGTTTCTTACTAGGTTGAGCAAGCATTTATATGTAAGAGCAGGCATTCTATTTCATTTGCGTTGTTAAG-GTGGCGCGAAGAGGACGAAACATACAGTTT--GTGATCAAGTATGTTATTGTAAAGAAATAATCATGGTAACAAGTAT-AATTCACGCATAATAATAGACGTTTAAGAGTATTTGTCTTT-AGGTGAAGT--ACTTGCATGGATCTATGAAATT-- 241

*** ***** ********* ***** * * * ********** **** ******** * * * **** *** * ****** ******** *** ** ************* ***** *** **************** *** ******* ***** ** **** ****** *

Consensus 90% -AAGGAsAAGGAsuus.uss.-ssssussssssGACTAGGTTGGGCAAGsuTTTs--------ussssGsATTCTATTTCsTTTGssTTGTTAAG-G-------ssGssssCussACATssAGTsT--usGATCAAGTATGTTAT-----usAAATAAT-ATGGTAACAAGTAs--sTTCACusATAATAATAGACGTTTAAGAuTATsTGTCTTT--GGTGAsGT--ssTTGCATGGATCsAsAuss----

80% -AAGGATAAGGAsuussGTss-TsuuAsuussTGACTAGGTTGGGCAAGCuTTTTss------AssssGsATTCTATTTCTTTTGCuTTGTTAAG-G-------TsGsTTsCuAuACATTsAGTsT--AsGATCAAGTATGTTAT-----GTAAATAAT-ATGGTAACAAGTAs--sTTCACATATAATAATAGACGTTTAAGAATATsTGTCTTT--GGTGAAGTTAuCTTGCATGGATCAAsAATs----

70% -AAGGATAAGGAsuusTGTCT-TuuuAsuuTsTGACTAGGTTGGGCAAGCuTTTTTs-----uAsCssGsATTCTATTTCTTTTGCuTTGTTAAGsG-------TsGTTTsCuAAACATTTAGTTT--AsGATCAAGTATGTTAT-----GTAAATAAT-ATGGTAACAAGTAs--sTTCACATATAATAATAGACGTTTAAGAATATsTGTCTTT--GGTGAAGTTAACTTGCATGGATCAATAATT----

60% -AAGGATAAGGAsAACTGTCT-TuGuACuGTTTGACTAGGTTGGGCAAGCuTTTTTs-----uAsCsTGTATTCTATTTCTTTTGCATTGTTAAGsG-------TTGTTTsCAAAACATTTAGTTT--AsGATCAAGTATGTTAT-----GTAAATAAT-ATGGTAACAAGTAs--ATTCACATATAATAATAGACGTTTAAGAATATsTGTCTTT-AGGTGAAGTTAACTTGCATGGATCAATAATT----

50% -AAGGATAAGGATAACTGTCT-TAGGACGGTTTGACTAGGTTGGGCAAGCATTTTTT-----AATCTTGTATTCTATTTCTTTTGCATTGTTAAGsG-------TTGTTTsCAAAACATTTAGTTT--ACGATCAAGTATGTTAT-----GTAAATAAT-ATGGTAACAAGTAA--ATTCACATATAATAATAGACGTTTAAGAATATsTGTCTTT-AGGTGAAGTTAACTTGCATGGATCAATAATT----

U68460 Z -AAGGACAAAGATAGCAATTTAGCTTTTTGCCAATCTAGGTTGAGCAAAC-CTTATGTTTCCTTGTCACTTCGCTTTTGCTGTCGAGATGGCGCAAATGTGTTTTCTTGACAGAAATTGAAAAGTT-----ATCAAGTTATCATAGATA-GTAAATAAATCTAGAAACAAGTACTCATTATCAAATCAATGGTGAATGGTATTCAATCGCATTTTTCCTGTGAGCTTTGCTCACGGGAGGAAAGAAAT

AGAGCT

GCAAAGCTGATTTGAATCAGCGCTTGA

TCGGTTGAATATCGATCAC

Consensus 90% -AAGGAsAAGGAsuus.uss--ssssussssssGACTAGGTTGGGCAAGsuTTTs--------ussssGsATTCTATTTCsTTTGssTTGTTAAG-G-------ssGssssCussACATssAGTsT--usGATCAAGTATGTTAT-----usAAATAAT-ATGGTAACAAGTAs--sTTCACusATAATAATAGACGTTTAAGAuTATsTGTCTTT--GGTGAsGT--ssTTGCATGGATCsAsAuss----

80% -AAGGATAAGGAsuussGTss-TsuuAsuussTGACTAGGTTGGGCAAGCuTTTTss------AssssGsATTCTATTTCTTTTGCuTTGTTAAG-G-------TsGsTTsCuAuACATTsAGTsT--AsGATCAAGTATGTTAT-----GTAAATAAT-ATGGTAACAAGTAs--sTTCACATATAATAATAGACGTTTAAGAATATsTGTCTTT--GGTGAAGTTAuCTTGCATGGATCAAsAATs----

70% -AAGGATAAGGAsuusTGTCT-TuuuAsuuTsTGACTAGGTTGGGCAAGCuTTTTTs-----uAsCssGsATTCTATTTCTTTTGCuTTGTTAAGsG-------TsGTTTsCuAAACATTTAGTTT--AsGATCAAGTATGTTAT-----GTAAATAAT-ATGGTAACAAGTAs--sTTCACATATAATAATAGACGTTTAAGAATATsTGTCTTT--GGTGAAGTTAACTTGCATGGATCAATAATT----

60% -AAGGATAAGGAsAACTGTCT-TuGuACuGTTTGACTAGGTTGGGCAAGCuTTTTTs-----uAsCsTGTATTCTATTTCTTTTGCATTGTTAAGsG-------TTGTTTsCAAAACATTTAGTTT--AsGATCAAGTATGTTAT-----GTAAATAAT-ATGGTAACAAGTAs--ATTCACATATAATAATAGACGTTTAAGAATATsTGTCTTT-AGGTGAAGTTAACTTGCATGGATCAATAATT----

50% -AAGGATAAGGATAACTGTCT-TAGGACGGTTTGACTAGGTTGGGCAAGCATTTTTT-----AATCTTGTATTCTATTTCTTTTGCATTGTTAAGsG-------TTGTTTsCAAAACATTTAGTTT--ACGATCAAGTATGTTAT-----GTAAATAAT-ATGGTAACAAGTAA--ATTCACATATAATAATAGACGTTTAAGAATATsTGTCTTT-AGGTGAAGTTAACTTGCATGGATCAATAATT----

AF193069 Bn9 -AAGGACAGGGATAACTG------AGGTTATCTTCCGAGGTTGGGCAAGCTCTATAAGCTTGACACATTGTCTTCTTTTCACTGTCGAATATCTAGAAAGGCTGATTGTAACAAATCAAGTA---------ATCAAAAAGCGTAAGATGAGCAAACAATAAAGTTTGCAAGTAAATAATCACTGATAAAAATCGACGCTTAAGAAGAATTCTTCATAAAATGGCGAAAGTCCGCAAGGACTATAATTT

TAA

AAA

AA

AAATCGAACTCTTATATTAAAGCTTGTTCCAATATAT

CAGTAAAAGTTTTTA

G

AA

Consensus 90% -AAGGAsAAGGAsuus.uss.-ssssussssssGACTAGGTTGGGCAAGsuTTTs--------ussssGsATTCTATTTCsTTTGssTTGTTAAG-G-------ssGssssCussACATssAGTsT--usGATCAAGTATGTTAT-----usAAATAAT-ATGGTAACAAGTAs--sTTCACusATAATAATAGACGTTTAAGAuTATsTGTCTTT--GGTGAsGT--ssTTGCATGGATCsAsAuss----

80% -AAGGATAAGGAsuussGTss-TsuuAsuussTGACTAGGTTGGGCAAGCuTTTTss------AssssGsATTCTATTTCTTTTGCuTTGTTAAG-G-------TsGsTTsCuAuACATTsAGTsT--AsGATCAAGTATGTTAT-----GTAAATAAT-ATGGTAACAAGTAs--sTTCACATATAATAATAGACGTTTAAGAATATsTGTCTTT--GGTGAAGTTAuCTTGCATGGATCAAsAATs----

70% -AAGGATAAGGAsuusTGTCT-TuuuAsuuTsTGACTAGGTTGGGCAAGCuTTTTTs-----uAsCssGsATTCTATTTCTTTTGCuTTGTTAAGsG-------TsGTTTsCuAAACATTTAGTTT--AsGATCAAGTATGTTAT-----GTAAATAAT-ATGGTAACAAGTAs--sTTCACATATAATAATAGACGTTTAAGAATATsTGTCTTT--GGTGAAGTTAACTTGCATGGATCAATAATT----

60% -AAGGATAAGGAsAACTGTCT-TuGuACuGTTTGACTAGGTTGGGCAAGCuTTTTTs-----uAsCsTGTATTCTATTTCTTTTGCATTGTTAAGsG-------TTGTTTsCAAAACATTTAGTTT--AsGATCAAGTATGTTAT-----GTAAATAAT-ATGGTAACAAGTAs--ATTCACATATAATAATAGACGTTTAAGAATATsTGTCTTT-AGGTGAAGTTAACTTGCATGGATCAATAATT----

50% -AAGGATAAGGATAACTGTCT-TAGGACGGTTTGACTAGGTTGGGCAAGCATTTTTT-----AATCTTGTATTCTATTTCTTTTGCATTGTTAAGsG-------TTGTTTsCAAAACATTTAGTTT--ACGATCAAGTATGTTAT-----GTAAATAAT-ATGGTAACAAGTAA--ATTCACATATAATAATAGACGTTTAAGAATATsTGTCTTT-AGGTGAAGTTAACTTGCATGGATCAATAATT----

AF042496 WSU861044-AA-GACATTCGCGAAAAGAT-------CTTCGCAAGAGGTTGAGCAAGCGGTCT-------------ATATTGC—-TTCCTTTACACTGTCAAGGCAGTGGTTTTGTTATCTTGAAAGTTTGAAT----GAT-AAGTATCAA-------GTAAAGTA-----GTAATCAGTAA----TCACTGAACTAAATCGACGCTTGAGGAAACAAATCCTTAAGAAAAAGTTAA----------------ATT----

A

CG

**Figure S4 B.**

16S promoter P2 region : chlamydial intergenic spacer

segment(88-113)

P2-10 ↓↓ **79**

CR848038 S26/3 CUUAACAAUGCAA**AU**GAAAUAGAAUG TATTCTATTTCTTTTGCATTGTTAAG

AE015925 GPIC CUUAACAAUGCAA**AU**GAGAUAGAAUG TATTCTATTTCTTTTGCATTGTTAAG

AP006861 Fe/C-56 DNA CUUAACAACGCAA**AU**GAGAUAGAAUG TATTCTATTTCTTTTGCGTTGTTAAG

AE009440 TWAR CUUAACAAUGCAA**AU**GAGAUAGAAUG CATTCTATTTCTTTTGCATTGTTAAG

CP000051 A/HAR-13 CUUAACAAUGCAA**AU**GAGAUAGAAUG CATTCTATTTCATTTGTGTTGTTAAG

AM884176 L2/434/BU CUUAACAAUGCAA**AU**GAGAUAGAAUG CATTCTATTTCATTTGTGTTGTTAAG

AE002160 MoPn CUUAACAAUGCAA**AU**GAGAUAGAAUG CATTCTATTTCATTTGCGTTGTTAAG
