# Supplementary figures and images for: Evolutionary Relationships among Chlamydophila abortus Variant Strains Inferred by rRNA Secondary Structure-Based Phylogeny
Source: PLoS One. 2011 May 24;6(5):e19813. doi: 10.1371/journal.pone.0019813 (PMC3101216; doi:10.1371/journal.pone.0019813)

**Figure S5 A.**


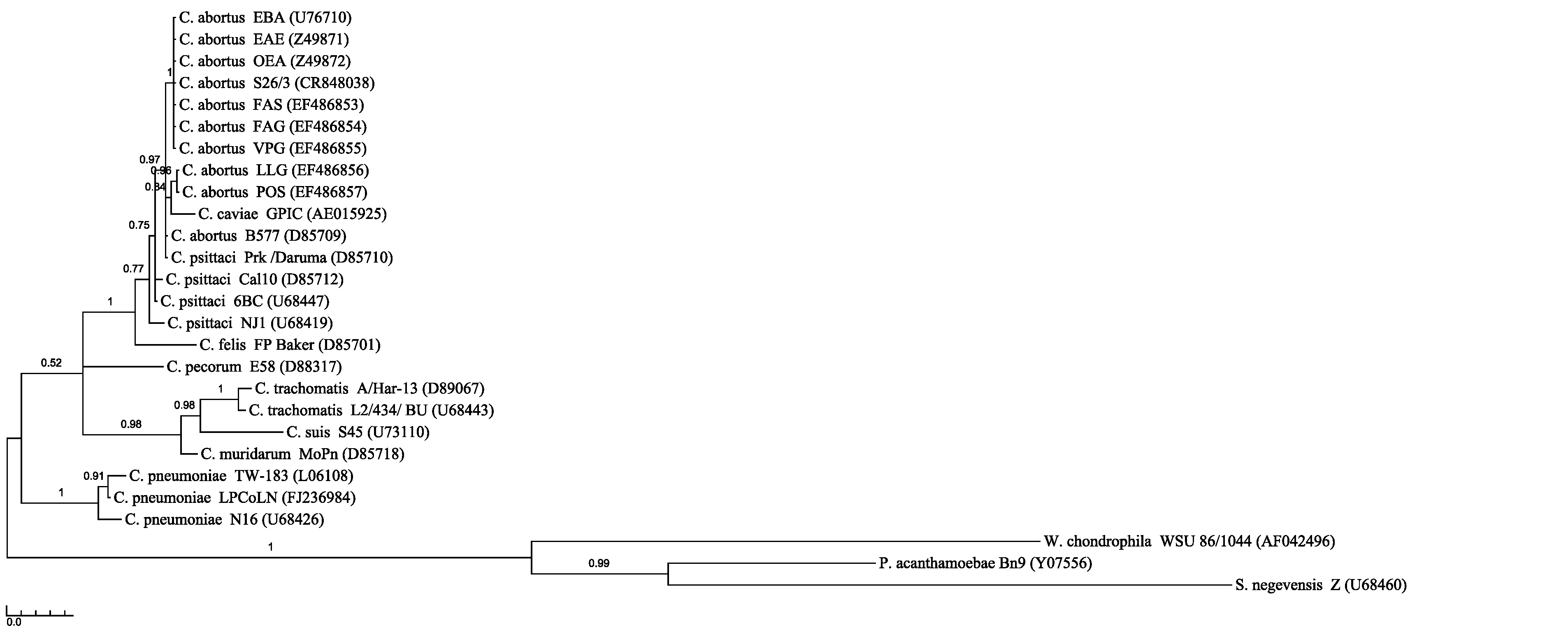


0.016

**Figure S5 B.**


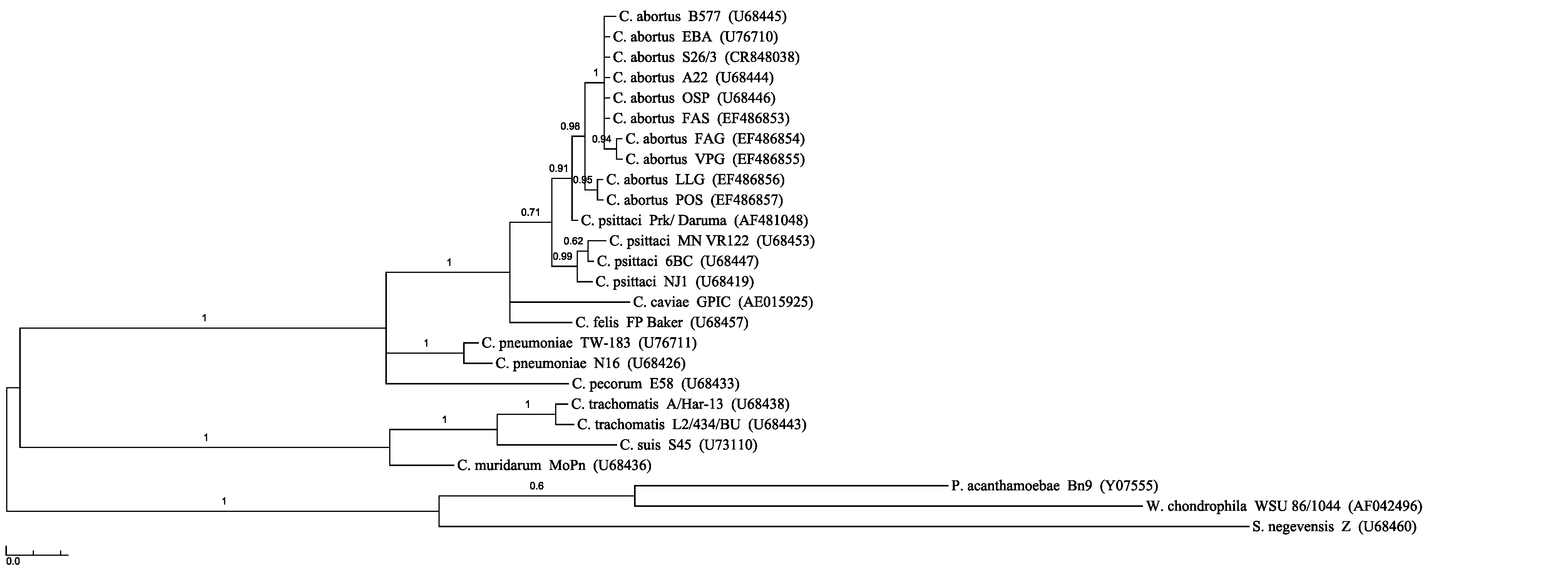
//: branches are shortened 2-fold.

**0.016**

**Figure S5 C.**


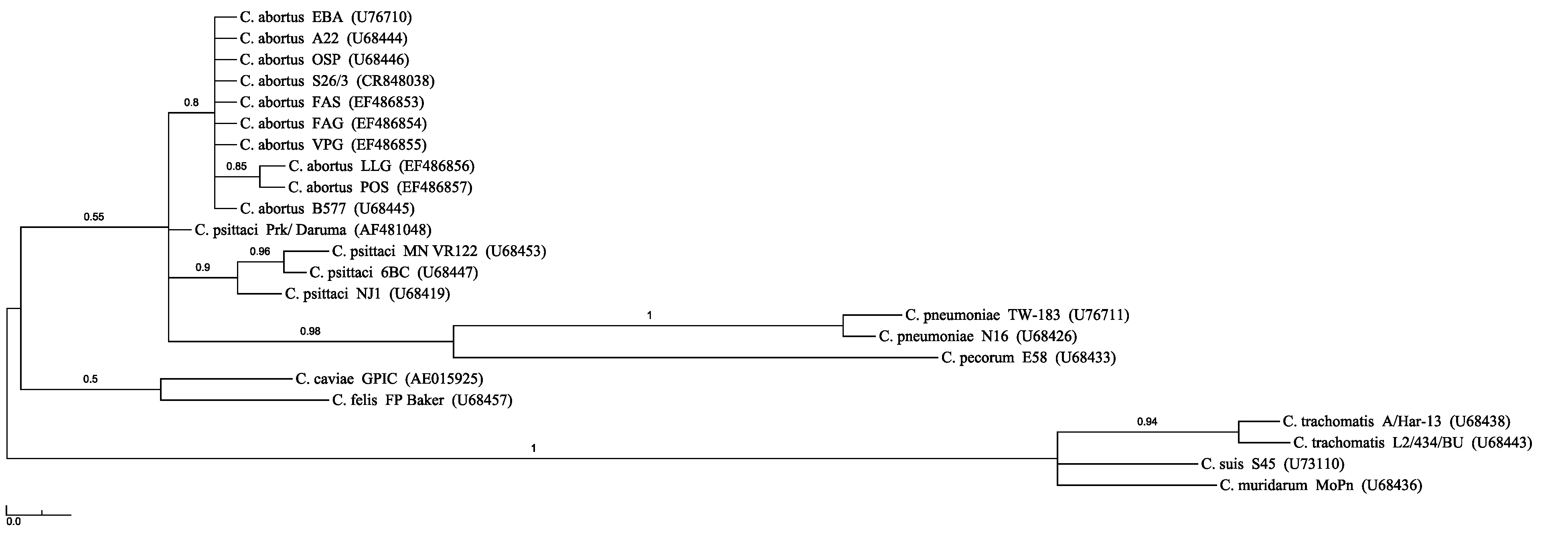
//: branches are shortened 2-fold.

**0.01**

Supplement: Figure S5 — Bayesian analysis (consensus trees) of 16S (A), 23S domain I (B), and 16S-23S IS (C) rRNA sequences of C. abortus strains and other Chlamydiales species. Numbers on branches indicate posterior probabilities. MrBayes version 3.1.2 was used [39]. The TreeGraph2 software [42] was used to display and manipulate the phylogenetic trees. (DOC) [file pone.0019813.s005.doc]
