# Supplementary material for: Evolutionary Relationships among Chlamydophila abortus Variant Strains Inferred by rRNA Secondary Structure-Based Phylogeny
Source: PLoS One. 2011 May 24;6(5):e19813. doi: 10.1371/journal.pone.0019813 (PMC3101216; doi:10.1371/journal.pone.0019813)
Supplement: Table S1 — Primer pairs and internal primer used for rDNA amplification and direct sequencing. (DOC) [file pone.0019813.s006.doc]

**Table S1.**

| **Primer** | **Positions*^a^*** | **Nucleotide sequence (5′ to 3′)** | **PCR (annealing temp. ° C) or sequencing targets** | **Reference or source** |
| --- | --- | --- | --- | --- |
| 16SFor | 1-20 (5-24)*^b^* | CTGAGAATTTGATCTTGGTT | 16S rRNA signature sequence (50) | This study |
| 16SIGR | 329-313 (325-309)*^b^* | TCAGTCCCAGTGTTGGC | 16S rRNA signature sequence | [2] |
|  |  |  |  |  |
| 16SF | 38-56 (42-60)*^b^* | GCGTGGATGAGGCATGCAA | Nearly full-length 16S rRNA (59) | [5] |
| 16SR | 1545-1528 (1539-1522)*^b^* | GGAGGTGATCCAGCCCCA |  | [5] |
|  |  |  |  |  |
| 16SF2 | 1403-1420 (1399-1416)*^b^* | CCGCCCGTCACATCATGG | 16S-23S rRNA spacer, 16S & 23S rRNA segments (48) | [30] |
| 23R | 1983-1965 (207-189)*^c^* | TACTAAGATGTTTCAGTTC |  | [30] |
|  |  |  |  |  |
| 16SF2 | 1403-1420 (1399-1416)*^b^* | CCGCCCGTCACATCATGG | 16S rRNA segment, 16S-23S spacer, 23S domain I (59) | [2] |
| 23SIGR | >2396-2385 (582-562)*^c^* | TGGCTCATCATGCAAAAGGCA |  | [2] |
|  |  |  |  |  |
| 409Rev | 1811-1792 (36-17)*^c^* | CCAAGGCATCCACCAATAAC | 16S-23S rRNA spacer, 16S rRNA segment | This study |

*^α^* The positions are given according to rRNA sequences determined in this study (GenBank accession numbers EF486853-EF486857). Numbers in parentheses are positions of the 16S (*^b^*) and 23S domain I (*^c^*)

rRNA genes according to *E. coli* numbering system.
