# Supplementary material for: Evolutionary Relationships among Chlamydophila abortus Variant Strains Inferred by rRNA Secondary Structure-Based Phylogeny
Source: PLoS One. 2011 May 24;6(5):e19813. doi: 10.1371/journal.pone.0019813 (PMC3101216; doi:10.1371/journal.pone.0019813)
Supplement: Table S2 — Comparison of VNTR and rRNA genotypes. (DOC) [file pone.0019813.s007.doc]

**Table S2.**

| **Strain** | **VNTR genotype*^a^*** | **rRNA genotype*^b^*** | **Allele no. at rRNA molecule*^b^*** | | |
| --- | --- | --- | --- | --- | --- |
|  |  |  | 16S rRNA | 23S rRNA domain I | 16S-23S IS |
| *C. abortus* B577^T^ (D85709, U68445) | [3] | A | 1 | 1*^c^* | 1 |
| *C. abortus*  LLG (EF486856) | [6] | B | 2 | 2 | 2 |
| *C. abortus*  POS (EF486857) | [6] | B | 2 | 2 | 2 |
| *C. abortus*  FAS (EF486853) | [5] | C | 3 | 3 | 1 |
| *C. abortus*  FAG (EF486854) | [2] | D | 3 | 4 | 1 |
| *C. abortus*  VPG (EF486855) | [2] | D | 3 | 4 | 1 |
| *C. abortus*  S26/3 (CR848038) | [5] | C | 3 | 3 | 1 |

*^a^* according to Laroucau et al. [17]

*^b^* For each rRNA molecule, the different sequences were assigned arbitrary allele numbers. For each strain, the combination of alleles obtained at each molecule defined its allelic profile. Therefore, each strain was designated by three numbers, constituting an allelic profile or rRNA genotype. We refer to a unique combination of alleles as an rRNA genotype. The rRNA genotypes were identified by arbitrary letters assigned in order of description.

*^c^* The 23S domain I of the *C. abortus* type strain B577^T^ sequence (acc. no. U68445) present the unique G538 character (*E. coli* numbering), not observed among other *Chlamydiales* members.
